# Abnormal coordination of executive control network and default mode network in cognitive impairment after deep nuclei intracerebral hemorrhage

**DOI:** 10.3389/fneur.2026.1915567

**Published:** 2026-09-16

**Authors:** Xiwen Fan, Yuchen Lei, Yongbing Deng, Yi Xiang, Chao Sun, Xing Yu, Jun Zhang, Chang Li, Peng Chen

**Affiliations:** 1 Department of Clinical Medicine, School of Medicine, Chongqing University, Chongqing, China; 2 Chongqing Key Laboratory of Emergency Medicine, Department of Neurosurgery, Chongqing Emergency Medical Center, Chongqing University Central Hospital, Chongqing, China; 3 Department of Health Management and Data Intelligence, Emlyon Business School and école des Mines de Saint-étienne, Lyon, France; 4 Department of Neurosurgery, Xinhua Hospital Affiliated to Shanghai Jiao Tong University School of Medicine, Shanghai, China; 5 Medical Imaging Department, Chongqing Emergency Medical Center, Chongqing University Central Hospital, Chongqing, China

**Keywords:** cognitive impairment, deep nuclei intracerebral hemorrhage, default mode network, executive control network, functional connectivity, spontaneous brain activity

## Abstract

**Objective:**

This study investigated the macroscopic neural mechanisms of cognitive impairment after Deep Nuclei Intracerebral Hemorrhage (DNICH) from the dimensions of brain activity and brain networks, and further identified characteristic fingerprints of cognitive representation.

**Method:**

Patients with cognitive impairment after DNICH and healthy controls (HC) were prospectively and consecutively enrolled. Resting-state fMRI (rs-fMRI) data and the cognitive scale Mini-Mental State Examination (MMSE) were collected. Spontaneous brain activity measured by fractional Amplitude of Low-Frequency Fluctuations (fALFF) was used to identify hub brain regions of cognitive representation, which were then used as Regions of Interest (ROI) for Functional Connectivity (FC) analysis, in order to investigate the potential mechanisms after DNICH cognitive impairment and identify characteristic fingerprints for distinguishing patients with dementia (dementia was defined as MMSE ≤ 17).

**Result:**

This study enrolled 139 patients with DNICH and 100 HC. There were no significant differences in baseline characteristics including age, gender, years of education, height, and weight between the two groups. Hematomas were predominantly located in the bilateral basal ganglia, more commonly on the left side, with varying degrees of thalamic and white matter involvement. Whole-brain zfALFF analysis showed that MMSE scores were significantly correlated with zfALFF values in brain regions including right dorsolateral prefrontal cortex (R-DLPFC) and posterior cingulate cortex (PCC), with R-DLPFC positively correlated and PCC negatively correlated with cognition (AlphaSim correction, *p* < 0.01; cluster size >19). In addition, FC within the executive control network (ECN) and default mode network (DMN) was abnormally altered in patients with cognitive impairment and was closely associated with cognitive function (*r* = −0.29–0.51). FC fingerprints demonstrated stable predictive ability for dementia status (AUC = 0.70–0.73).

**Conclusion:**

Abnormal coordination of intra-network and inter-network FC of ECN and DMN may represent a shared neural mechanism of cognitive impairment after DNICH. Modulating FC between these hub brain regions (R-DLPFC and PCC) may provide new therapeutic strategies for cognitive recovery after DNICH.

## Introduction

Stroke imposes an enormous socioeconomic burden globally. Intracerebral hemorrhage (ICH) was one of the most common types of strokes, accounting for 28.8% of all stroke subtypes (1). ICH imposed a disproportionately high disease burden, accounting for 45.6% of stroke-related deaths and 49.6% of total disability-adjusted life years (DALYs) (2). Despite advances in stroke management, ICH remained a leading cause of mortality and long-term disability worldwide, highlighting the urgent need for enhanced preventive and therapeutic strategies. The deep nuclei involving the basal ganglia region encompassed critical structures, including the putamen, caudate nucleus, globus pallidus, thalamus and internal capsule, etc. Lesions in these structures not only disrupt motor-sensory pathways, leading to the classic triad syndrome, but also impair executive function through dysregulation of cognitive control networks, resulting in a clinical phenotype with combined motor and cognitive deficits.

Advances in medical care have improved acute-phase survival after ICH, leading to a growing population of survivors. Cognitive impairment after ICH became increasingly common, with reported incidences of 37.7–71.2% in the acute phase and 27–42.7% in the chronic phase. Notably, cognitive impairment affected 35.9–55.9% of survivors after Deep Nuclei Intracerebral Hemorrhage (DNICH), representing a substantial burden on functional independence and quality of life (3). However, the mechanisms underlying cognitive impairment after DNIH remained incompletely understood.

Functional Magnetic Resonance Imaging (fMRI) was a noninvasive neuroimaging technique that relied on Blood-Oxygen-Level-Dependent (BOLD) signals. By detecting neuronal activity–induced changes in cerebral blood flow, blood volume, and oxygen metabolism, fMRI allowed dynamic monitoring of brain function and Functional Connectivity (FC), serving as a principal tool for investigating cognitive processes. Our previous study indicated that resting-state fMRI (rs-fMRI) demonstrated that the medial prefrontal cortex (mPFC) and left parahippocampal gyrus within the default mode network (DMN) were critically involved in cognitive regulation after basal ganglia intracerebral hemorrhage (BGIH) (4).

This study employed rs-fMRI to identify hub brain regions associated with cognitive impairment after DNICH. And the study further analyzed brain network FC changes based on these regions compare with healthy controls (HC) to provide brain network theoretical evidence for understanding mechanisms of cognitive impairment after DNICH.

## Method

### Participants

This prospective, observational study was conducted in accordance with the Strengthening the Reporting of Observational Studies in Epidemiology (STROBE) statement (5). The individuals diagnosed with ICH, who were admitted to the Neurosurgery Department between January 2024 and January 2026, were eligible for inclusion.

**DNICH Inclusion criteria** (4): (1) Age between 18 and 85 years; (2) Radiological confirmation of DNICH (The deep nuclei involving the basal ganglia region encompassed critical structures, including the putamen, caudate nucleus, globus pallidus, thalamus and internal capsule, etc.,) within 24 h of symptom onset via computed tomography (CT) or magnetic resonance imaging (MRI); (3) MRI performed within 24 h after clinical scale assessment; (4) Right-handed; (5) Normal pre-onset abilities in communication, work, and activities of daily living; (6) Stable vital signs and ability to breathe spontaneously.

**Exclusion criteria** (4): (1) Coexistent psychiatric disorders or other neurological conditions; (2) Prior history of significant head injury, stroke, or intracranial neoplasm; (3) Presence of severe concurrent systemic illness; (4) Documented history of alcohol misuse, substance use disorder, or illicit drug use; (5) Absolute or relative contraindications to MRI scanning (including cardiac pacemaker, metallic implants, or severe claustrophobia); (6) Current pregnancy; (7) Persistent inability to remain still or cooperate sufficiently for MRI acquisition despite 2–3 repeated attempts.

Additionally, 100 healthy participants were selected from the research team's database to serve as the control group, whose baseline characteristics were broadly comparable to those of the current DNICH cohort.

### Clinical data collection

At baseline, the following information was recorded: demographic characteristics, time interval from symptom onset to functional imaging acquisition, lesion laterality, Mini-Mental State Examination (MMSE), and National Institutes of Health Stroke Scale (NIHSS) score. The study referenced previously published research, defining MMSE ≤ 17 as dementia and MMSE > 17 as non-dementia (6, 7).

Importantly, NIHSS scores were assessed on admission. rs-fMRI scans were performed either immediately before or within 24 h after the administration of MMSE scales. This timing protocol was implemented to ensure that functional imaging data closely reflected the patients' cognitive status at the time of assessment.

### rs-fMRI acquisition

All neuroimaging examinations were performed using a 3.0 Tesla MRI scanner (Discovery MR750; GE Healthcare) equipped with an 8-channel phased-array head coil. The rs-fMRI were acquired with a gradient-echo echo-planar imaging sequence using the following parameters (4): repetition time (TR)/echo time (TE) = 2,000/30 ms, flip angle = 70°, field of view = 240 × 240 mm, matrix size = 64 × 64, slice thickness = 3.5 mm, and 33 axial slices acquired in interleaved order to cover the whole brain. Each functional run consisted of 240 volumes obtained over an 8-min scanning period.

High-resolution structural images were collected using a three-dimensional T1-weighted BRAVO sequence with these parameters (4): TR/TE = 8.16/3.18 ms, flip angle = 12°, matrix = 256 × 256, thickness = 1 mm, and 168 slices spanning the entire brain.

### Lesion frequency mapping

A lesion overlap frequency map was generated using a standardized and precise approach. Lesion delineation was performed using MRIcroGL software (https://www.nitrc.org/search/?type_of_search=group&q=mricrogl). The detailed procedure included the following steps: (1) Manual segmentation of the hematoma on each relevant slice, with the segmented DNICH volume recorded and stored as part of the baseline; (2) The post-segmentation T1-weighted image (with lesion masked out) was saved for rs-fMRI preprocessing; (3) Normalization of the individual lesion masks into standard Montreal Neurological Institute (MNI) space; (4) Resampling of the normalized lesion masks to an isotropic resolution of 1 × 1 × 1 mm^3^; (5) Construction of a lesion frequency map that maintains hemispheric laterality, calculated by overlaying all normalized lesion masks onto a common template and counting the number of overlapping lesions at each voxel (where “a” represents the lesions mask, “b” the whole-brain template mask, and “c” the whole-brain mask after subtraction of the lesions mask).

### rs-fMRI pre-processing

Preprocessing of the rs-fMRI data was performed using the DPABI V7.0 toolbox (http://rfmri.org/DPABI) within MATLAB R2016b (MathWorks, Natick, MA, USA). The pipeline comprised the following sequential steps: (1) Conversion of raw DICOM files to NIFTI format; (2) Discarding the initial 10 volumes to allow for magnetic field stabilization; (3) Correction for differences in slice acquisition timing; (4) Realignment to correct for head movement, with exclusion of participants exhibiting excessive motion (translational displacement > 2.0 mm or rotational displacement > 2° in any direction); (5) Co-registration of high-resolution structural T1-weighted images to the mean functional image to ensure accurate alignment between anatomical and functional data; (6) Tissue segmentation and spatial normalization to MNI template space using the Diffeomorphic Anatomical Registration Through Exponentiated Lie algebra (DARTEL) toolbox; (7) Regression of nuisance covariates prior to normalization, including the white matter signal, cerebrospinal fluid signal, and Friston 24-parameter model of head motion; (8) Removal of linear trends across the time series; (9) Application of temporal bandpass filtering within the 0.01–0.1 Hz range to retain hemodynamically relevant low-frequency oscillations; (10) Spatial smoothing of the normalized functional volumes with an isotropic Gaussian kernel of 4 × 4 × 4 mm full width at half maximum to improve signal-to-noise ratio while minimizing residual spatial noise.

### rs-fMRI post-processing

Whole-brain fractional amplitude of low-frequency fluctuations (fALFF) was computed after excluding signals from bilateral lesions, followed by z-score transformation to improve normality (z-fALFF). Correlation analyses between zfALFF and MMSE were performed, adjusting for age, gender, education, hemorrhage laterality, and hemorrhage volume, to identify brain regions representing cognitive activity after DNICH (AlphaSim correction, *p* < 0.01; cluster size > 19 voxels). These cognitive-representative regions served as regions of interest (ROIs) for whole-brain FC analysis after excluding bilateral lesion signals, followed by z-transformation (zFC), and were compared with healthy controls using two-sample t-tests with lesion “c” mask (AlphaSim correction, *p* < 0.01; cluster size > 19 voxels). Subsequently, the cognitive-representative ROIs were employed for whole-brain FC analysis after excluding bilateral lesion signals, followed by zFC, and correlated with MMSE while adjusting for confounders (age, gender, education, hemorrhage laterality, and hemorrhage volume) to identify regions representing cognitive FC (AlphaSim correction, *p* < 0.01; cluster size > 19 voxels). FC values of the identified regions (the average across multiple significant clusters) were subsequently extracted and subjected to correlation analysis with adjustment for confounders. Finally, zFC values were utilized to distinguish between dementia and non-dementia DNICH groups.

### Statistical analysis

The conventional statistical analyses were performed using SPSS version 26 (https://www.ibm.com/products/spss-statistics) and GraphPad Prism 9.5 (https://www.graphpad.com/). The Kolmogorov-Smirnov test was used to assess normality. Normally distributed data were presented as mean ± standard deviation, while non-normally distributed data were reported as median and interquartile range. For the data with normal distribution, the two-sample t-test was used for intergroup comparison. For the data with non-normal distribution, the rank sum test was employed. For the binary data, the chi-square test was applied.

## Result

### Demographics and clinical data

A total of 708 participants with ICH admitted to our center between January 2024 and 2026 were initially screened for study eligibility. Excluded were 241 participants with aneurysmal subarachnoid hemorrhage or cerebral amyloid angiopathy–related intracerebral hemorrhage. We further excluded participants aged < 18 or >85 years, or those with onset time>24 h (*n* = 57). Additionally, 248 participants with ICH of non- basal ganglia region were excluded. Following the exclusion of 23 participants due to incomplete clinical data or missing essential information, a final cohort of 139 participants with DNICH (Figure 1) were included in the final analysis.

**Figure 1:**
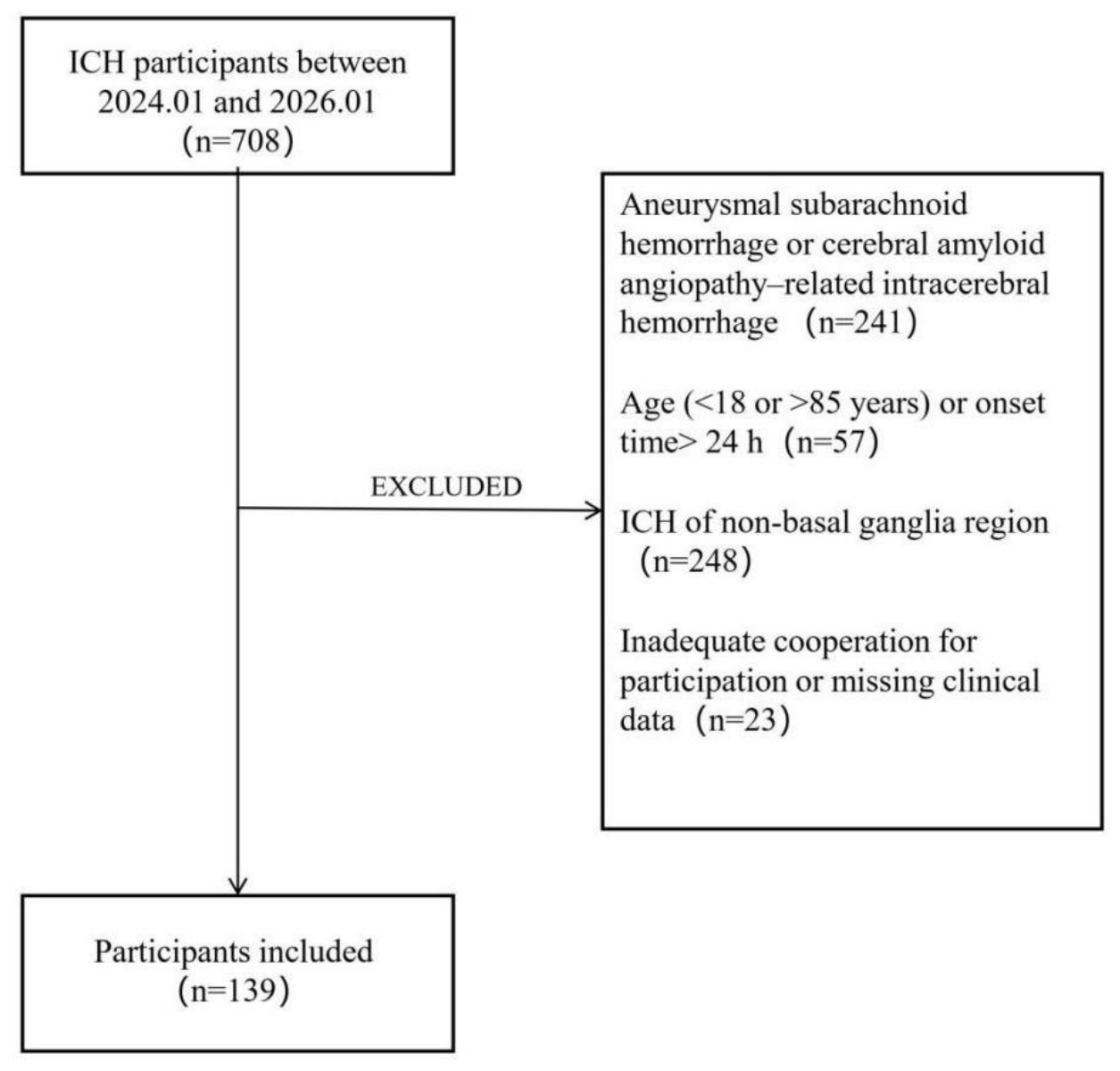
DNICH participants screening flowchart.

The study included 139 participants with DNICH involving the basal ganglia region. The mean age of the participants was 56.09 ± 12.88 years, 57.6% were male, and all were right-handed. The mean years of education was 8.58 ± 3.87 years, mean height was 167.09 ± 8.37 cm, and mean weight was 69.47 ± 15.43 kg. Hematoma was predominantly located on the left side in 78 cases (56.1%), and the median hematoma volume was 16.5 ml. The median NIHSS score at admission was 12.00, the median time interval from admission to MMSE assessment was 11 days, and the median MMSE score was 18.00.

The study also included 100 HC, with a mean age of 55.5 ± 13.62 years, 53% male, and all were right-handed. The mean years of education was 8.73 ± 3.55 years, mean height was 167.39 ± 7.68 cm, and mean weight was 67.75 ± 11.88 kg. There were no significant differences in baseline characteristics such as age, gender, years of education, height, and weight between patients with HC and DNICH. The baseline data of the study participants were detailed in Table 1.

**Table 1:** Baseline and scale characteristics of participants.

Characteristics	HC *n* = 100	DNICH *n* =139	T/*U*/χ2-value	*P-*value
**Age**	55.50 ± 13.62	56.09 ± 12.88	0.34	0.73
**Gender, male (%)**	53 (53.00)	80 (57.60)	0.32	0.57
**Right-handedness (%)**	100 (100.00)	139 (100.00)	-	-
**Education, years**	8.73 ± 3.55	8.58 ± 3.87	0.32	0.75
**Height, cm**	167.39 ± 7.68	167.09 ± 8.37	0.28	0.78
**Weight, kg**	67.75 ± 11.88	69.47 ± 15.43	0.98	0.33
**Lesion side, left**	-	78 (56.10)	-	-
**Lesion volume, ml**	-	16.50 (6.34–26.56)	-	-
**Onset to investigation interval, d**	-	11.00 (7.00–19.00)	-	-
**NIHSS**	-	12.00 (5.00–20.00)	-	-
**MMSE**	-	18.00 (5.00–25.00)	-	-

HC, healthy controls; DNICH, deep nuclei intracerebral hemorrhage; Normally distributed data: mean ± standard deviation; non-normal data: median and its quartiles; cm, centimeter; kg, kilogram; ml, milliliter; d, day; NIHSS, National Institutes of Health Stroke Scale; MMSE, Mini-Mental State Examination.

We stratified the 139 enrolled patients into two subgroups using an MMSE cutoff score of 17: 73 patients had MMSE scores > 17, and 66 patients had MMSE scores ≤ 17. Consistent with the diagnostic criteria referenced in our manuscript, patients with MMSE scores > 17 were categorized as the non-dementia group, while those with scores ≤ 17 were assigned to the dementia group. Statistically significant intergroup differences were identified in age, years of education, NIHSS scores, and hematoma volume, with full comparison results presented in Table 2. The core factor to be considered was the hematoma volume. Larger hematoma volumes were linked to greater clinical complexity, prolonged recovery, higher NIHSS scores, and longer intervals between admission and cognitive assessment.

**Table 2:** Baseline and scale characteristics of dementia and non-dementia patients.

Characteristics	Dementia *n* = 66	Non-dementia *n* = 73	T/*U*/χ2-value	*P-*value
**Age**	58.55 ± 12.53	53.88 ± 12.88	2.16	0.03
**Gender, male (%)**	40 (60.61)	40 (54.79)	0.48	0.49
**Right-handedness (%)**	66 (100.00)	73 (100.00)	-	-
**Education, years**	7.71 ± 3.01	9.36 ± 4.38	−2.55	0.01
**Height, cm**	167.97 ± 8.74	166.30 ± 7.99	1.18	0.24
**Weight, kg**	70.47 ± 15.51	68.58 ± 15.40	0.72	0.47
**Lesion side, left (%)**	42 (63.63)	36 (49.32)	2.89	0.09
**Lesion volume, ml**	21.05 (9.67–29.79)	12.00 (4.55–23.65)	3.11	0.002
**Onset to investigation interval, d**	15.00 (10.00–22.25)	9.00 (6.00–14.50)	3.67	< 0.001
**NIHSS**	20.00 (14.00–24.00)	8.00 (2.00–10.50)	8.22	< 0.001
**MMSE**	5.00 (2.00–10.00)	25.00 (22.00–27.00)	−10.18	< 0.001

Normally distributed data: mean ± standard deviation; non-normal data: median and its quartiles; cm, centimeter; kg, kilogram; ml, milliliter; d, day; NIHSS, National Institutes of Health Stroke Scale; MMSE, Mini-Mental State Examination.

### Spatial distribution characteristics of hematoma

To analyze the spatial distribution pattern of hematomas, we segmented and superimposed MRI images from 139 participants. The hematoma overlay map was shown in Figure 2. The results showed that the hemorrhage was mainly concentrated in the basal ganglia region, including the lentiform nucleus and internal capsule, with a relatively equal distribution on the left and right sides. In some participants, the hematoma also involved the thalamus, corona radiata, and periventricular white matter.

**Figure 2:**
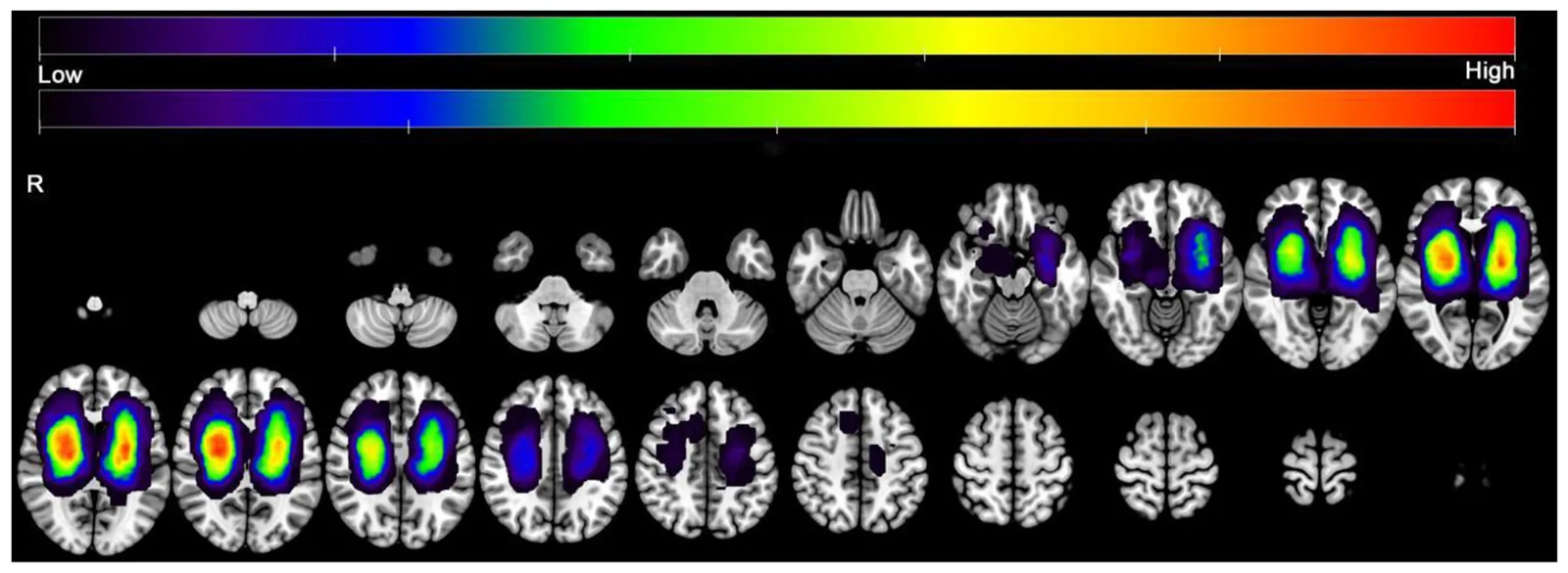
Spatial distribution of hematomas in DNICH participants.

The lesion overlaps frequency map displayed hematoma locations across 139 participants. The color gradient (from cool to warm tones) represented overlap frequency from low to high.

### Whole-brain zfALFF analysis of brain regions associated with cognition in DNICH

After adjusting for variables including age, gender, years of education, hemorrhage side, and hemorrhage volume, we analyzed the regional correlation between whole-brain zfALFF and MMSE scores. The results showed significant correlations between MMSE scores and multiple brain regions (AlphaSim correction, *p* < 0.01, cluster size > 19 voxels; Supplementary Table 1 and Figure 3).

**Figure 3:**
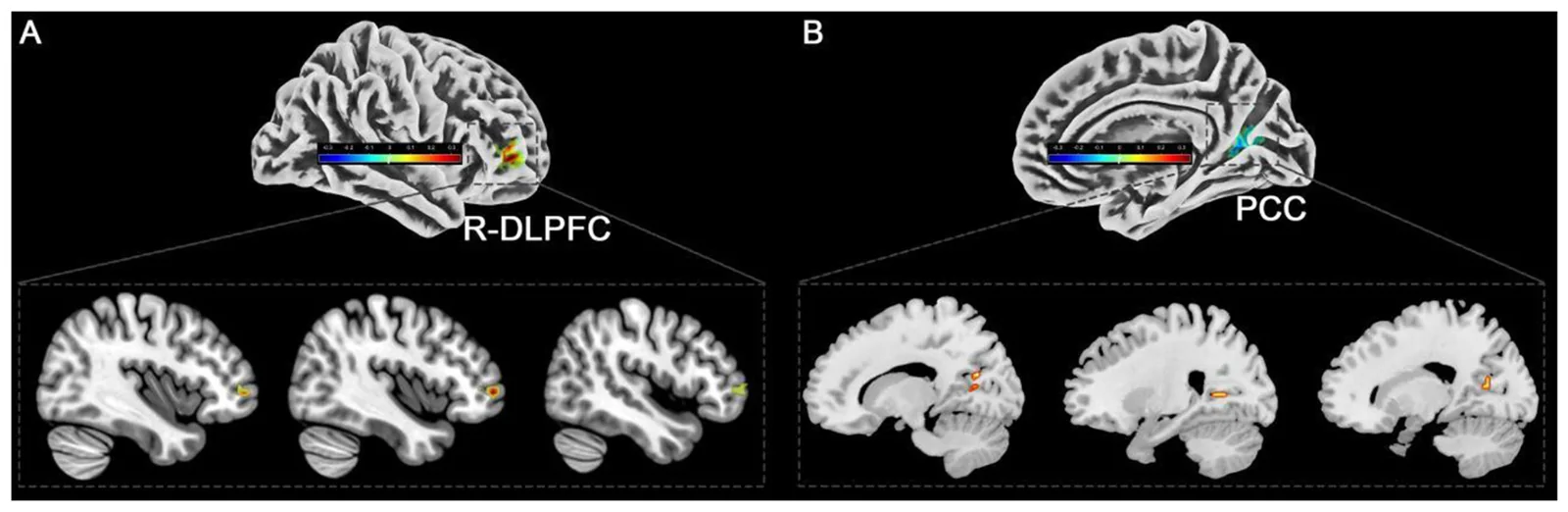
Brain regions showed significant correlations between zfALFF and cognitive performance. (**A**) Regions with positive correlations between zfALFF values and MMSE scores. (**B**) Regions with negative correlations between zfALFF values and MMSE scores. The “r” denotes the correlation coefficient; The color gradient (from cool to warm tones) represented correlation coefficients from low to high. Statistical threshold: AlphaSim correction *p* < 0.01, cluster size >19 voxels. R-DLPFC, right dorsolateral prefrontal cortex; PCC, posterior cingulate cortex.

Among these, brain regions showing a positive correlation between zfALFF and cognition were mainly located in the right frontal lobe, with the typical region being Right Dorsolateral Prefrontal Cortex (R-DLPFC); brain regions showing a negative correlation between zfALFF and cognition were mainly located in Posterior Cingulate Cortex (PCC).

### Executive Control Network (ECN): comparison of R-DLPFC ROI-seeded FC between DNICH patients and HC

Considering that R-DLPFC and PCC serve as the core hubs of the ECN and DMN, respectively, and that these two networks maintain healthy cognitive function through dynamic and flexible connectivity. Therefore, we used R-DLPFC as ROI to construct ECN and compared it with the ECN of HC; we also used PCC as ROI to construct DMN and compared it with DMN of HC, in order to explore the brain network mechanisms of cognitive impairment in patients with DNICH.

The ECN constructed with R-DLPFC as ROI was compared with that of 100 HC (ECN constructed after removing bilateral lesion areas, Supplementary Figure 1), and the difference map was obtained (AlphaSim correction, *p* < 0.01, cluster size > 19 voxels). The results were as follows:

The red regions represented brain areas where FC of R-DLPFC was enhanced in DNICH patients compared with HC, including: medial prefrontal lobe, temporal lobe, limbic lobe, etc. Typical brain regions included: Brodmann areas 8, 9, 10, 11, 32, middle temporal gyrus, Brodmann area 21, fusiform gyrus, calcarine sulcus, cuneus, lingual gyrus, posterior cingulate, and parahippocampal gyrus, etc., (Figure 4A, Supplementary Table 2).

**Figure 4:**
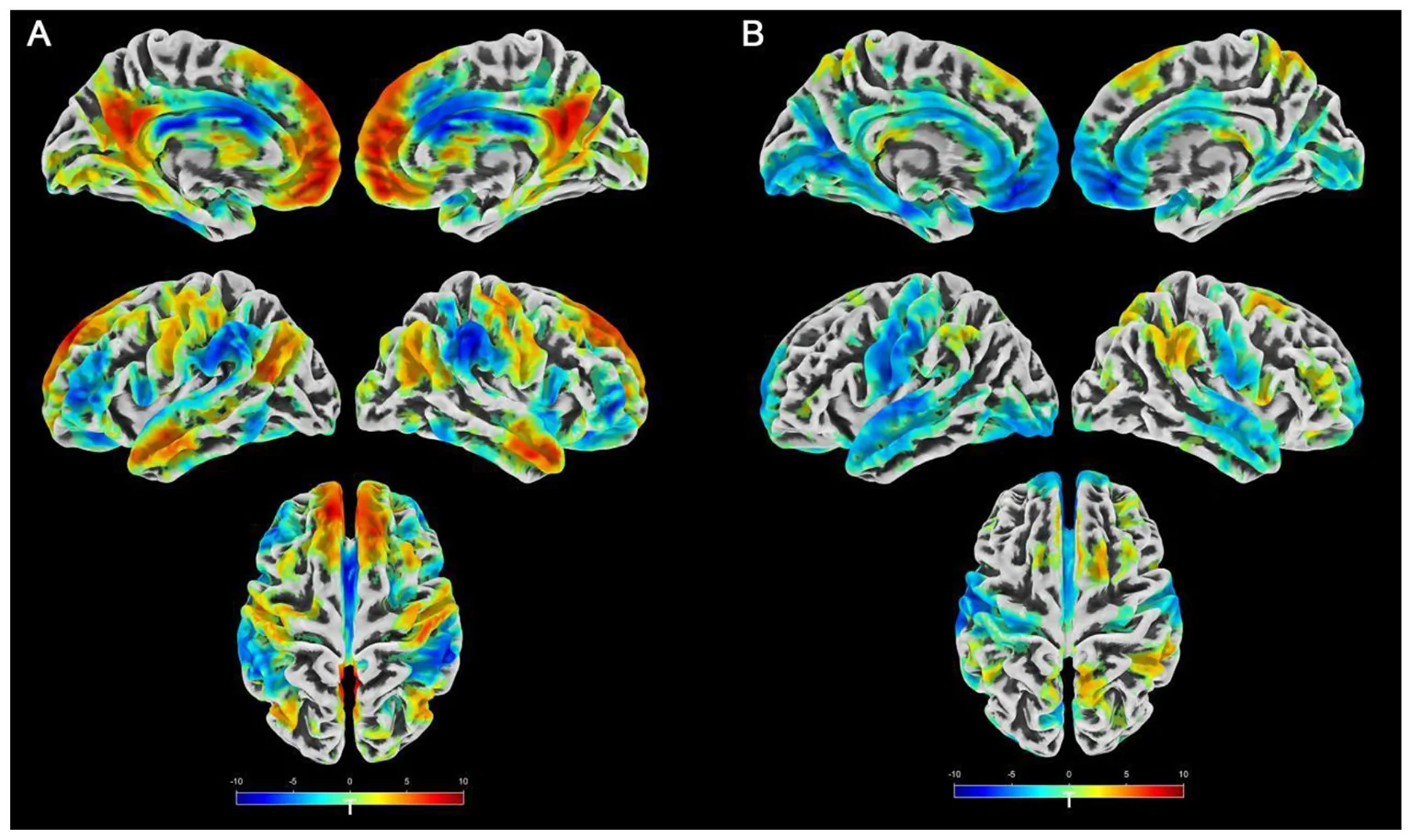
DNICH patients compared with HC, maps of ECN with R-DLPFC (**A**) as ROI and DMN with PCC (**B**). The color gradient (from cool to warm tones) represented FC strength from low to high.

The blue regions represent brain areas where FC with R-DLPFC was decreased after DNICH compared with HC (AlphaSim correction, *p* < 0.01, cluster size > 19 voxels), including: lateral middle and inferior frontal gyrus, Brodmann areas 44, 45, 47, inferior parietal lobule, superior temporal gyrus, Brodmann area 40, angular gyrus, and anterior cingulate (Figure 4A, Supplementary Table 2).

### Default Mode Network (DMN): comparison of PCC ROI-seeded FC between DNICH patients and HC

Based on 139 patients with DNICH, we used PCC as ROI to construct DMN and compared it with DMN of 100 HC (the control network was constructed after removing bilateral lesion areas, Supplementary Figure 2), and the difference map was obtained (AlphaSim correction, *p* < 0.01, cluster size > 19 voxels). The results showed as follows.

The red regions represent brain areas where FC with PCC was enhanced after DNICH compared with HC, including: frontal lobe, parietal lobe, occipital lobe. Typical brain regions included: middle frontal gyrus, Brodmann area 47, Brodmann area 6, 7, angular gyrus, cuneus, lingual gyrus, and Brodmann area 18, etc. (Figure 4B, Supplementary Table 3).

The blue regions represent brain areas where FC with PCC was decreased after DNICH compared with HC, including: medial prefrontal lobe, temporal lobe, parietal lobe. Typical brain regions include: Brodmann areas 10, 11, 12, superior temporal gyrus, middle temporal gyrus, parahippocampal gyrus, calcarine sulcus, anterior cingulate, and postcentral gyrus (Figure 4B, Supplementary Table 3).

### Brain regions with cognitive-correlated FC using R-DLPFC and PCC as ROIs in DNICH patients

Brain regions where FC constructed with R-DLPFC as ROI positively correlated with cognition (AlphaSim correction, *p* < 0.01, cluster size > 19 voxels) included: frontal lobe, temporal lobe. Typical brain regions were: left middle frontal gyrus, right inferior frontal gyrus, superior temporal gyrus, left middle temporal gyrus, left fusiform gyrus, and left parahippocampal gyrus (Figure 5A, Supplementary Table 4).

**Figure 5:**
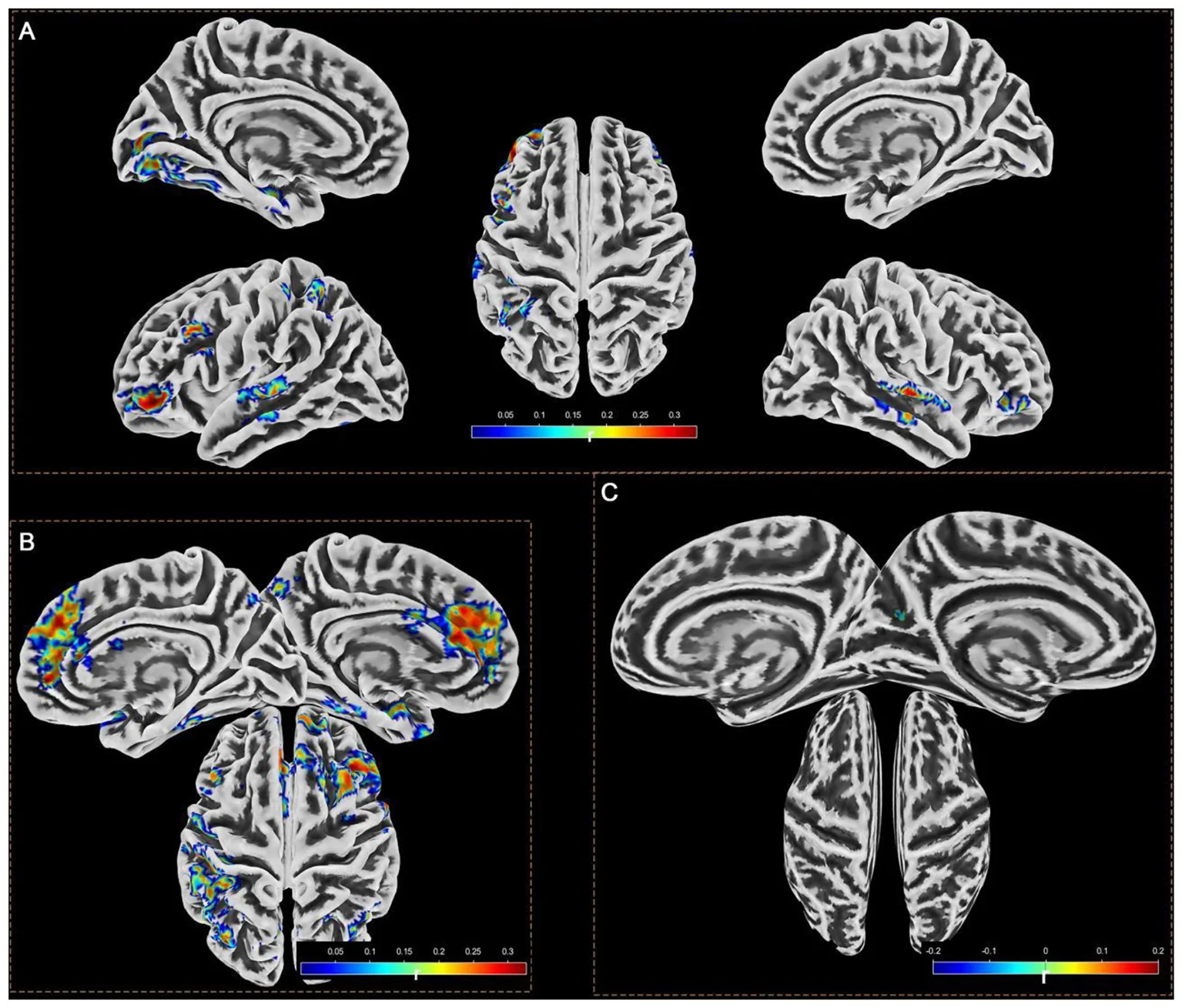
Brain region maps of FC with R-DLPFC and PCC as ROI correlated with cognition. (**A**) Brain region map of FC with R-DLPFC as ROI positively correlated with cognition. (**B**) Brain region map of FC with PCC as ROI positively correlated with cognition. (**C**) Brain region map of FC with PCC as ROI negatively correlated with cognition. The “r” denotes the correlation coefficient; The color gradient (from cool to warm tones) represented correlation coefficients from low to high.

Brain regions where FC constructed with PCC as ROI positively correlated with cognition (AlphaSim correction, *p* < 0.01, cluster size > 19 voxels) included: frontal lobe, temporal lobe, parietal lobe, occipital lobe. Typical brain regions included: medial frontal gyrus, Brodmann areas 9, 10, 32, anterior cingulate, limbic lobe, superior temporal gyrus, middle temporal gyrus, Brodmann areas 21, 22, right fusiform gyrus, postcentral gyrus, inferior parietal lobule, Brodmann areas 2, 3, 40, precuneus, and angular gyrus (Figure 5B, Supplementary Table 5).

In addition, the only brain region where FC constructed with PCC as ROI negatively correlated with cognition was the left cuneus (AlphaSim correction, *p* < 0.01, cluster size > 19 voxels, Figure 5C, Supplementary Table 5).

### Quantitative analysis of zFC values in different brain regions and cognitive levels

For further quantitative analysis, we extracted the zFC values in different brain regions of Figure 5, and performed correlation analysis with MMSE scores. The zFC values in differential brain regions of Figures 5A and B shown in Figures 6A and B were significantly positively correlated with cognitive scores, with statistical significance (Figures 6A, B). The zFC values in differential brain regions of Figure 5C shown in Figure 6C were significantly negatively correlated with cognitive scores, with statistical significance (Figure 6C).

**Figure 6:**
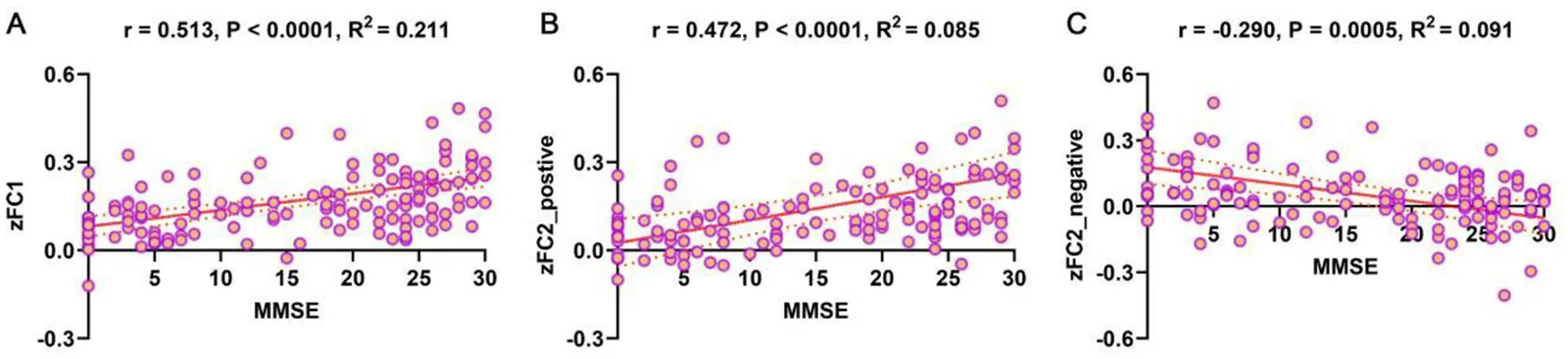
Correlation analysis between zFC values in different brain regions and MMSE scores. (**A**) zFC1 values in brain regions of Figure 5A were statistically significantly positively correlated with MMSE scores (*r* = 0.513, *p* < 0.0001, *R*^2^ = 0.211). (**B**) zFC2 values in brain regions of Figure 5B were statistically significantly positively correlated with MMSE scores (*r* = 0.472, *p* < 0.0001, *R*^2^ = 0.085). (**C**) zFC3 values in brain regions of Figure 5C were statistically significantly negatively correlated with MMSE scores (*r* = −0.290, *p* = 0.0005, *R*^2^ = 0.091).

### Ability of differential FC brain regions to predict dementia status

We also extracted the zFC values in different brain regions of Figure 5 to perform an exploratory ROC analysis for identifying the subgroup of DNICH patients with MMSE ≤ 17. The demographics and clinical data of the patient cohort have been presented in Table 2. The results showed that the areas under the ROC curves (AUCs) for the three brain regions were 0.70, 0.73, and 0.71, respectively, indicating moderate discriminative ability (Figures 7A–C). These diagnostic performance results represented preliminary, hypothesis-generating evidence rather than validated clinical diagnostic efficacy. Nevertheless, they demonstrated encouraging discriminative capacity and held notable promise as candidate imaging biomarkers for further investigation.

**Figure 7:**
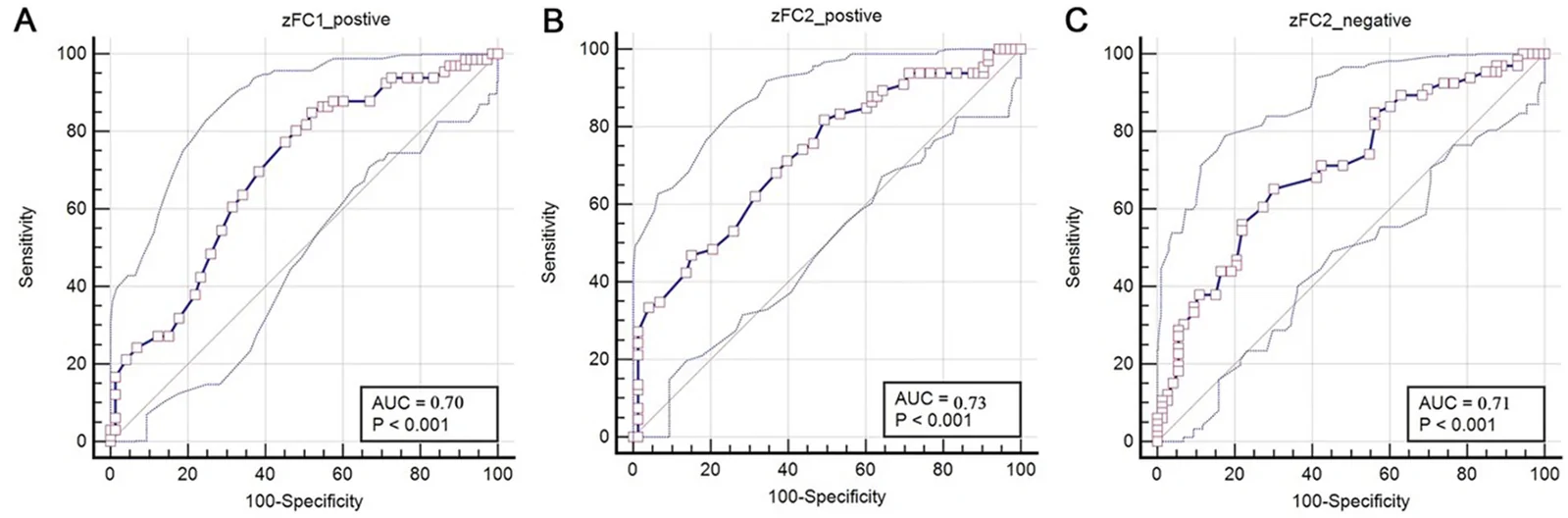
ROC curves for zFC values in different brain regions to identify MMSE ≤ 17. (**A**) Ability of FC maps (R-DLPFC as ROI, positively correlated with cognition) to identify patients with dementia. (**B**) Ability of FC maps (PCC as ROI, positively correlated with cognition) to identify patients with dementia. (**C**) Ability of FC maps (PCC as ROI, negatively correlated with cognition) to identify patients with dementia.

## Discussion

Cognitive impairment was a major complication following stroke that significantly impairs patients' activities of daily living, reduces quality of life, and increases the risk of post-stroke mortality. The incidence of cognitive impairment after DNICH was as high as 35.9–55.9% (3). However, the neural network mechanisms, early-warning fingerprints, and neuromodulation targets for cognitive impairment after DNICH remain unclear. fMRI owing to its non-invasive nature and high spatiotemporal resolution, was an effective tool for investigating the neural mechanisms of cognitive impairment. To address this critical knowledge gap, the present study utilized rs-fMRI to delineate the functional network mechanisms underlying cognitive impairment after DNICH. This study also aimed to identify imaging biomarkers of cognitive decline and propose candidate neuromodulation targets, with the ultimate goal of improving clinical management for this high-risk patient population.

We first found that the brain regions in which zfALFF was positively correlated with cognition were predominantly located in the right frontal lobe, with R-DLPFC as the representative region. The brain regions in which zfALFF was negatively correlated with cognition were predominantly PCC. Previous studies have demonstrated that structural and functional alterations of the DLPFC and PCC, as the core hub nodes of large-scale cognitive networks, were significantly associated with post-stroke cognitive impairment (8, 9). In cognitive-related disorders including Alzheimer's disease (AD) and Parkinson's disease (PD), structural and functional disruption of the DLPFC and PCC cortical association areas constituted a key neural basis for the development of cognitive impairment (10–12). Extensive evidence highlighted that cognitive impairment across different diseases shared similar neuropathological network pathways. Additionally, our previous research found left parahippocampal gyrus, the left superior temporal gyrus, right temporal regions, the cerebellum, and posterior cingulate cortex exhibited significant correlation with cognitive impairment and gene functional decoding analyses revealed strong association with chemical synaptic transmission, synaptic signaling, and cognitive processing (4).

We consider that functional connectivity of DLPFC, PCC were correlated with cognitive function, and DLPFC and PCC were key regions of the ECN and DMN, respectively. The DLPFC was primarily responsible for working memory, attentional regulation, and executive function, whereas the PCC played a central role in internally directed thought, memory retrieval, and regulation of attentional breadth and balance. The ECN and DMN form a dynamic equilibrium through functional antagonistic coupling: the ECN governed externally driven cognitive processing and goal-directed behavior, while the DMN supported internally generated mental activity and self-referential processing. They maintained the homeostatic transition of brain function between task execution and resting-state introspection (13). Current research has primarily shown that disruption of default network white matter tract integrity, along with reduction in Regional Homogeneity (ReHo) and FC within its constituent regions, was critical to the development of post-stroke cognitive impairment (14–16). However, studies investigating the antagonistic relationship between ECN and DMN from a large-scale brain network perspective remained limited. In particular, the patterns of network reorganization after DNICH and their role in post-hemorrhagic cognitive impairment have not been fully characterized (4). Further investigation into the neural brain network fingerprints and neuromodulation targets of cognitive impairment after DNICH was therefore warranted to provide a theoretical basis for clinical treatment.

This study used R-DLPFC and PCC as ROI to construct the ECN and DMN, and compared them with the corresponding brain networks in HC. The ECN core region R-DLPFC showed reduced FC in intra-network regions including the anterior cingulate cortex and inferior parietal lobule, and increased FC with DMN regions including the medial prefrontal cortex and PCC. The DMN core region PCC also showed reduced FC in intra-network medial prefrontal cortex, and increased FC with ECN regions including R-DLPFC and the inferior parietal lobule.

The enhanced positive correlation between ECN and DMN observed in this study indicated that the two networks, which were normally antagonistic, underwent dedifferentiation. This aberrant coupling reduced signal discriminability, impairing the brain's ability to distinguish between introspective and attentional states, thereby resulting in reduced cognitive efficiency. This mechanism has been identified in post-stroke disorders of consciousness, where enhanced direct connectivity between DLPFC and PCC following stroke contributes to cognitive decline (8). Changes in static and dynamic inter-network connectivity among the ECN, DMN, and Salience Network also constituted a mechanism underlying cognitive impairment (17–19). Reduced FC between ECN and DMN has been reported in the early post-stroke period, indicating that inter-network functional impairment and compensation coexist and co-evolve across different disease stages, which accounts for the complexity of post-stroke cognitive impairment mechanisms (20). Therefore, our zFC analyses in DNICH patients demonstrated that subcortical hemorrhage drove pathological remodeling of large-scale cognitive networks, characterized by aberrantly increased inter-network FC between ECN and DMN, coupled with compromised integrity of canonical intra-network connections within each network. This loss of physiological reciprocal antagonism between the two core cognitive networks drove rigid network dynamics and impaired rest-task state transitions, ultimately providing a robust, network-level mechanistic rationale for the progressive cognitive deterioration observed after DNICH.

The structure governed function, and the reconfiguration of functional attributes rose from alterations in structure. Hematoma inflicted physical structural damage to deep gray matter nuclei and the white matter fiber tracts, directly interrupting the neural conduction pathways between DMN and ECN, and triggering connectivity disturbance and functional reorganization of networks (4, 21). Mounting evidence indicated that subcortical hemorrhagic lesions, even when restricted to the basal ganglia or thalamus, could cause extensive and asymmetric cortical gray matter atrophy in remote brain regions, including the hippocampus, fusiform gyrus, lateral temporal cortex, prefrontal cortex, somatomotor cortex, and inferior parietal lobule (22). Meanwhile, ICH resulted in profound structural and functional damage to the cerebral microvasculature, triggering a cascade of hemodynamic abnormalities including reduced regional cerebral perfusion and impaired cerebrovascular autoregulation (4). These microcirculatory disturbances subsequently led to secondary hypoxic-ischemic and metabolic insults in distant cognition-related cortical regions, most notably DLPFC and PCC (23, 24). These secondary remote injuries further destabilized intrinsic low-frequency neural oscillations and aggravated aberrant spontaneous neural activity within key cortical regions. Consequently, the functional integrity of brain networks was substantively impaired. Such disturbances ultimately contributed to the onset and progression of cognitive impairment after DNICH (25, 26). In addition, as a pivotal hub of the cortico-striato-thalamo-cortical circuit, the basal ganglia exhibited an imbalance in the synthesis and transmission of cognition-related neurotransmitters after DNICH. This abnormality gave rise to aberrant neural oscillations in the circuit and ultimately mediated network dysfunction dominated by DLPFC and PCC, which served as a crucial anatomical and circuit basis for cognitive impairment (23, 24, 27). These pathophysiological manifestations after DNICH were highly consistent with the findings of our current study.

Further prognostic analyses were performed for dementia status. The results demonstrated that models constructed based on zFC values of key brain regions yielded AUC values of 0.70, 0.73, and 0.71, respectively, exhibiting favorable discriminative performance for dementia after DNICH. Previous studies have confirmed that dementia risk prediction predominantly relies on models established by integrating multimodal data with sophisticated machine learning algorithms, with their discriminative AUC values generally ranging from 0.70 to 0.85 (28–30). In this study, comparable discriminative efficacy was achieved using only a single neuroimaging metric, namely zFC values derived from rs-fMRI, conferring remarkable advantages for clinical application. Nevertheless, it is worth noting that NIHSS score, hemorrhage side, and time from onset may potentially influence FC, and subsequently modulate cognitive performance. Our team has further investigated the cognitive effects of hemorrhagic lateralization in a separate study. Preliminary results reveal that hemorrhage induces shared and lateralized alterations in FC. Notably, left-sided DNICH may lead to more severe cognitive impairment. This univariate imaging model not only validated the central role of FC associated with DLPFC and PCC in the pathogenesis of cognitive impairment after DNICH, but also confirmed its potential as a neuroimaging biomarker. Considering the limitations of existing cognitive assessment tools for DNICH, our research group could subsequently conduct studies integrating neuroimaging metrics, clinical scales, and biological biomarkers to construct a multidimensional comprehensive prognostic system, to further improve the accuracy of early prediction for cognitive impairment after DNICH.

### Limitation

This study has several limitations. First, the study analyzed the potential mechanism of cognitive impairment after DNICH only at the level of FC networks, and was unable to provide clarification at the level of brain structure and structure-function coupling. Second, although the sample size of the DNICH study reached 139 cases, whether multicenter data could provide stable support for the current findings remains to be investigated. Third, although this study proposed that aberrant coupling between ECN and DMN was the core mechanism of cognitive impairment after DNICH, the causal relationship between the strength of FC between ECN and DMN and cognitive level could not be established. Fourth, the potential coupling characteristics of ECN and DMN were identified based on overall cognitive dimensions, and whether these characteristic fingerprints were shared across individual cognitive domains remained unknown.

## Conclusion

Our study found that FC between R-DLPFC and DMN brain regions was enhanced, while FC with ECN brain regions was reduced. Similarly, PCC showed enhanced FC with ECN brain regions and reduced FC with DMN brain regions. Intra-network FC within each independent network thus declined, whereas inter-network FC was increased. The breakdown of intra-network FC within the ECN and DMN, alongside this aberrant inter-network coupling, may account for the cognitive impairment observed after DNICH. Modulating both intra- and inter-network FC through the hub regions of these two networks, R-DLPFC and PCC held promise for improving cognitive outcomes after DNICH.

## Data Availability

The original contributions presented in the study are included in the article/Supplementary material, further inquiries can be directed to the corresponding author/s.
