# Supplementary material for: Abnormal coordination of executive control network and default mode network in cognitive impairment after deep nuclei intracerebral hemorrhage
Source: Front Neurol. 2026 Sep 16;17:1915567. doi: 10.3389/fneur.2026.1915567 (PMC13623672; doi:10.3389/fneur.2026.1915567)
Supplement: Supplementary file 2 [file Data_Sheet_2.pdf]

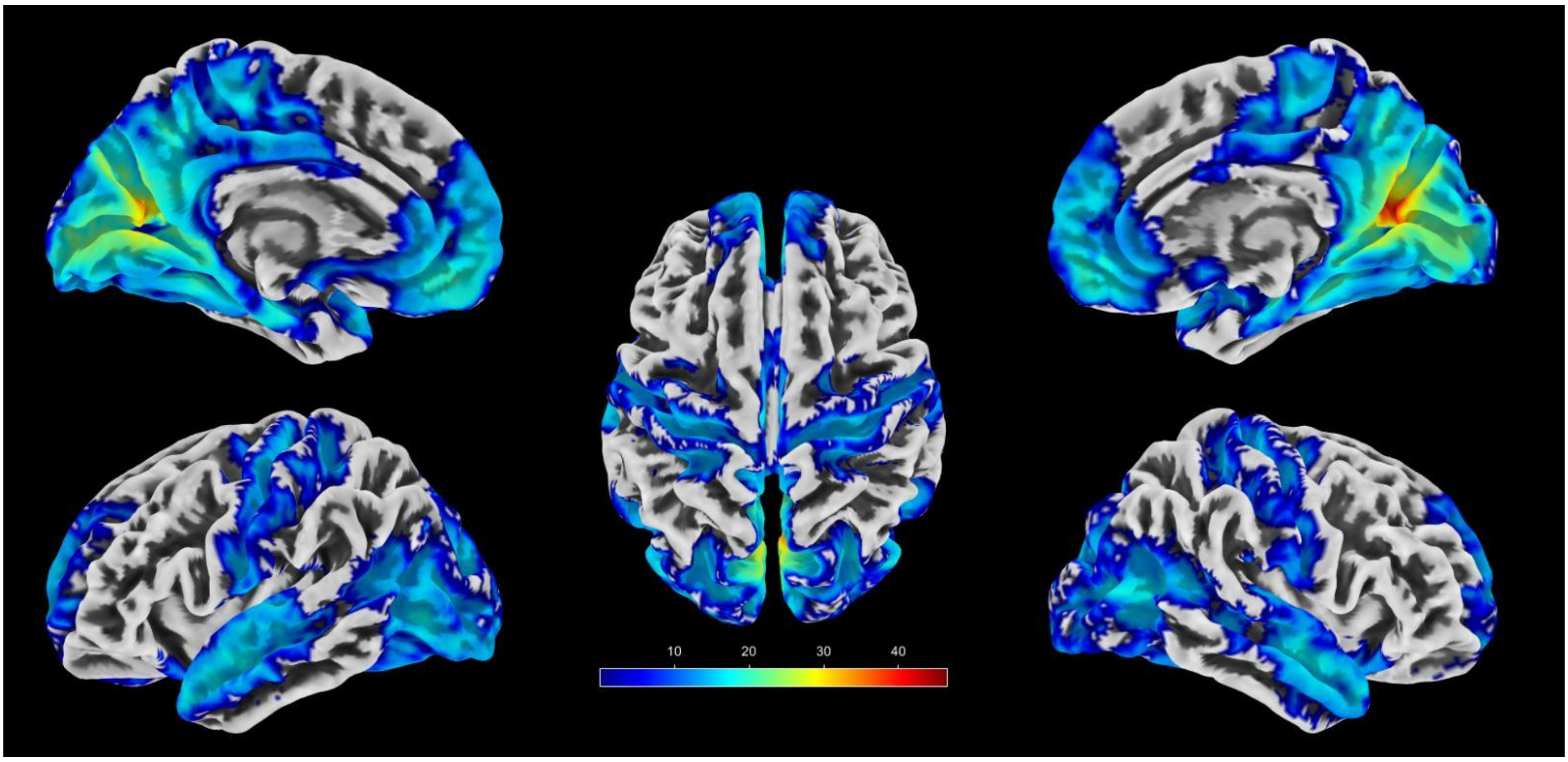

The functional connectivity map (default mode network) was created using the right posterior cingulate cortex as the region of interest, involving 100 healthy controls, with bilateral lesion areas excluded and T-values represented by a color bar.
