# Supplementary material for: Abnormal coordination of executive control network and default mode network in cognitive impairment after deep nuclei intracerebral hemorrhage
Source: Front Neurol. 2026 Sep 16;17:1915567. doi: 10.3389/fneur.2026.1915567 (PMC13623672; doi:10.3389/fneur.2026.1915567)
Supplement: Supplementary file 3 [file Table_1.docx]

Supplementary Table 1 **Brain detail regions showed significant correlations between zfALFF and cognitive performance**

This report is based on CUI Xu's xjview. (http://www.alivelearn.net/xjview/)

Revised by YAN Chao-Gan and ZHU Wei-Xuan 20091108: suitable for different Cluster Connectivity Criterion: surface connected, edge connected, corner connected.

Number of clusters found: 2

----------------------

Cluster 1

Number of voxels: 24

Peak MNI coordinate: 42 51	0

Peak MNI coordinate region: // Right Cerebrum // Frontal Lobe // Middle Frontal Gyrus // White Matter // undefined // Frontal_Mid_R (aal)

Peak intensity: 0.38732

\# voxels	structure

24	--TOTAL # VOXELS--

24	Frontal Lobe

24	Right Cerebrum

20	Frontal_Mid_R (aal)

14	White Matter

13	Middle Frontal Gyrus

10	Gray Matter

10	Inferior Frontal Gyrus

9	brodmann area 10

2	Frontal_Inf_Tri_R (aal)

2	Frontal_Mid_Orb_R (aal)

1	Sub-Gyral

----------------------

Cluster 2

Number of voxels: 22

Peak MNI coordinate: 15 -66 21

Peak MNI coordinate region: // Right Cerebrum // Parietal Lobe // Precuneus // White Matter // undefined // Precuneus_R (aal)

Peak intensity: -0.27239

\# voxels	structure

22	--TOTAL # VOXELS--

22	Right Cerebrum

19	White Matter

17	Calcarine_R (aal)

7	Posterior Cingulate

7	Limbic Lobe

6	Precuneus

6	Sub-lobar

5	Parietal Lobe

5	Precuneus_R (aal)

5	Extra-Nuclear

3	Sub-Gyral

3	Occipital Lobe

2	brodmann area 30

2	Gray Matter

1	Lateral Ventricle

1	Cerebro-Spinal Fluid

1	Temporal Lobe

>>
