# Supplementary material for: Abnormal coordination of executive control network and default mode network in cognitive impairment after deep nuclei intracerebral hemorrhage
Source: Front Neurol. 2026 Sep 16;17:1915567. doi: 10.3389/fneur.2026.1915567 (PMC13623672; doi:10.3389/fneur.2026.1915567)
Supplement: Supplementary file 4 [file Table_2.docx]

Supplementary Table 2 **Compared with HC, brain detail regions of ECN with R-DLPFC as ROI**

This report is based on CUI Xu's xjview. (http://www.alivelearn.net/xjview/)

Revised by YAN Chao-Gan and ZHU Wei-Xuan 20091108: suitable for different Cluster Connectivity Criterion: surface connected, edge connected, corner connected.

Number of clusters found: 16

----------------------

Cluster 1

Number of voxels: 114

Peak MNI coordinate: 6 -54 -48

Peak MNI coordinate region: // Right Cerebellum // Cerebellum Posterior Lobe // Cerebellar Tonsil // undefined // undefined // Cerebelum_9_R (aal)

Peak intensity: 5.7211

\# voxels	structure

114	--TOTAL # VOXELS--

92	Cerebellum Posterior Lobe

75	Cerebellar Tonsil

59	Cerebelum_9_R (aal)

54	Right Cerebellum

40	Left Cerebellum

31	Cerebelum_9_L (aal)

16	Inferior Semi-Lunar Lobule

3	Cerebelum_8_L (aal)

2	Medulla

2	Right Brainstem

1	Nodule

1	Cerebellum Anterior Lobe

1	Cerebro-Spinal Fluid

1	Sub-lobar

1	Uvula of Vermis

1	Vermis_9 (aal)

1	Fourth Ventricle

----------------------

Cluster 2

Number of voxels: 492

Peak MNI coordinate: -57 -6 -21

Peak MNI coordinate region: // Left Cerebrum // Temporal Lobe // Inferior Temporal Gyrus // Gray Matter // brodmann area 21 // Temporal_Mid_L (aal)

Peak intensity: 7.2636

\# voxels	structure

492	--TOTAL # VOXELS--

464	Left Cerebrum

456	Temporal Lobe

338	Temporal_Mid_L (aal)

291	Middle Temporal Gyrus

258	White Matter

162	Gray Matter

113	brodmann area 21

68	Temporal_Inf_L (aal)

58	Inferior Temporal Gyrus

49	Temporal_Pole_Mid_L (aal)

40	Fusiform Gyrus

39	Superior Temporal Gyrus

28	Sub-Gyral

26	brodmann area 20

21	brodmann area 38

11	Fusiform_L (aal)

8	Temporal_Pole_Sup_L (aal)

7	Limbic Lobe

5	Parahippocampa Gyrus

2	brodmann area 22

1	Sub-lobar

1	Lateral Ventricle

1	Hippocampus_L (aal)

1	Temporal_Sup_L (aal)

1	Cerebro-Spinal Fluid

----------------------

Cluster 3

Number of voxels: 421

Peak MNI coordinate: 57	6 -27

Peak MNI coordinate region: // Right Cerebrum // Temporal Lobe // Middle Temporal Gyrus // Gray Matter // brodmann area 21 // Temporal_Mid_R (aal)

Peak intensity: 7.7191

\# voxels	structure

421	--TOTAL # VOXELS--

345	Temporal Lobe

345	Right Cerebrum

193	Temporal_Mid_R (aal)

193	Middle Temporal Gyrus

167	Gray Matter

131	White Matter

130	Temporal_Pole_Mid_R (aal)

101	brodmann area 21

72	Temporal_Inf_R (aal)

63	Superior Temporal Gyrus

57	Inferior Temporal Gyrus

40	brodmann area 38

26	brodmann area 20

25	Fusiform Gyrus

10	Temporal_Pole_Sup_R (aal)

8	Temporal_Sup_R (aal)

6	Sub-Gyral

----------------------

Cluster 4

Number of voxels: 42

Peak MNI coordinate: 27 -81 -33

Peak MNI coordinate region: // Right Cerebellum // Cerebellum Posterior Lobe // Uvula // undefined // undefined // Cerebelum_Crus1_R (aal)

Peak intensity: 4.8142

\# voxels	structure

42	--TOTAL # VOXELS--

42	Right Cerebellum

42	Cerebellum Posterior Lobe

28	Cerebelum_Crus1_R (aal)

25	Uvula

14	Declive

14	Cerebelum_Crus2_R (aal)

3	Tuber

----------------------

Cluster 5

Number of voxels: 3597

Peak MNI coordinate: 12 60	0

Peak MNI coordinate region: // Right Cerebrum // Frontal Lobe // Medial Frontal Gyrus // White Matter // undefined // Frontal_Sup_Medial_R (aal)

Peak intensity: 8.8136

\# voxels	structure

3597	--TOTAL # VOXELS--

2682	Frontal Lobe

1509	White Matter

1496	Left Cerebrum

1427	Right Cerebrum

1278	Superior Frontal Gyrus

1226	Gray Matter

928	Medial Frontal Gyrus

530	Frontal_Sup_Medial_L (aal)

512	Frontal_Sup_Medial_R (aal)

460	Frontal_Sup_L (aal)

369	Frontal_Sup_R (aal)

349	brodmann area 10

270	Middle Frontal Gyrus

259	brodmann area 8

248	Inter-Hemispheric

247	brodmann area 9

180	Limbic Lobe

180	Anterior Cingulate

158	brodmann area 11

142	Frontal_Med_Orb_L (aal)

142	Rectus_L (aal)

140	Frontal_Mid_R (aal)

129	Rectus_R (aal)

128	Supp_Motor_Area_L (aal)

110	Frontal_Med_Orb_R (aal)

104	Frontal_Mid_L (aal)

100	Cingulum_Ant_L (aal)

91	Cingulum_Ant_R (aal)

83	brodmann area 6

76	Sub-Gyral

64	Sub-lobar

56	brodmann area 32

41	Frontal_Sup_Orb_L (aal)

38	Rectal Gyrus

37	Frontal_Inf_Orb_L (aal)

35	Orbital Gyrus

30	Inferior Frontal Gyrus

24	Lateral Ventricle

24	Cerebro-Spinal Fluid

23	Caudate

18	Caudate_L (aal)

18	Frontal_Sup_Orb_R (aal)

17	Extra-Nuclear

17	Supp_Motor_Area_R (aal)

16	brodmann area 25

16	Caudate Head

14	Olfactory_R (aal)

9	brodmann area 46

7	Caudate Body

7	brodmann area 47

7	Frontal_Inf_Tri_R (aal)

5	Corpus Callosum

5	brodmann area 24

3	Frontal_Mid_Orb_L (aal)

3	Frontal_Inf_Tri_L (aal)

3	Subcallosal Gyrus

2	Caudate_R (aal)

2	Olfactory_L (aal)

1	Cingulum_Mid_R (aal)

1	Thalamus_L (aal)

----------------------

Cluster 6

Number of voxels: 232

Peak MNI coordinate: 12 -96 -21

Peak MNI coordinate region: // Right Cerebrum // Occipital Lobe // Lingual Gyrus // Gray Matter // brodmann area 17 // undefined

Peak intensity: 4.3212

\# voxels	structure

232	--TOTAL # VOXELS--

231	Right Cerebrum

215	Occipital Lobe

158	White Matter

106	Cuneus

104	Calcarine_R (aal)

67	Gray Matter

45	Lingual Gyrus

42	Middle Occipital Gyrus

27	brodmann area 17

27	Sub-Gyral

22	Lingual_R (aal)

18	brodmann area 18

16	Occipital_Sup_R (aal)

13	brodmann area 19

11	Temporal Lobe

11	Occipital_Mid_R (aal)

10	Cuneus_R (aal)

9	Fusiform_R (aal)

9	Occipital_Inf_R (aal)

6	Temporal_Inf_R (aal)

4	Sub-lobar

4	Inferior Temporal Gyrus

2	Lateral Ventricle

2	Extra-Nuclear

2	Cerebro-Spinal Fluid

1	Posterior Cingulate

1	Precuneus

1	Limbic Lobe

1	brodmann area 23

1	brodmann area 37

1	Inferior Occipital Gyrus

----------------------

Cluster 7

Number of voxels: 1892

Peak MNI coordinate: 3 -51 18

Peak MNI coordinate region: // Right Cerebrum // Limbic Lobe // Posterior Cingulate // Gray Matter // brodmann area 30 // Precuneus_R (aal)

Peak intensity: 8.7689

\# voxels	structure

1892	--TOTAL # VOXELS--

973	Left Cerebrum

887	White Matter

779	Limbic Lobe

692	Right Cerebrum

568	Gray Matter

406	Posterior Cingulate

357	Precuneus

342	Occipital Lobe

307	Parietal Lobe

280	Precuneus_L (aal)

238	Precuneus_R (aal)

215	Parahippocampa Gyrus

184	Calcarine_L (aal)

171	Lingual Gyrus

167	Lingual_L (aal)

148	Sub-Gyral

140	Cingulate Gyrus

139	brodmann area 31

124	Temporal Lobe

123	Sub-lobar

104	Calcarine_R (aal)

103	Culmen

103	Cerebellum Anterior Lobe

99	Fusiform_L (aal)

93	Inter-Hemispheric

89	Lingual_R (aal)

86	brodmann area 7

85	brodmann area 30

84	Extra-Nuclear

82	Cingulum_Post_L (aal)

65	Left Cerebellum

65	Cuneus_L (aal)

62	Cuneus

60	Fusiform_R (aal)

59	Cingulum_Post_R (aal)

54	Corpus Callosum

45	Cerebelum_4_5_L (aal)

43	ParaHippocampal_R (aal)

42	brodmann area 29

41	brodmann area 19

41	brodmann area 18

39	Cerebro-Spinal Fluid

39	Lateral Ventricle

38	Right Cerebellum

37	Vermis_4_5 (aal)

34	brodmann area 23

34	Cingulum_Mid_L (aal)

33	Fusiform Gyrus

28	brodmann area 36

28	brodmann area 37

27	ParaHippocampal_L (aal)

23	Occipital_Sup_L (aal)

23	Cerebelum_4_5_R (aal)

20	Hippocampus_R (aal)

18	Cuneus_R (aal)

16	Occipital_Mid_L (aal)

15	Cingulum_Mid_R (aal)

13	brodmann area 17

10	Occipital_Inf_L (aal)

7	Middle Occipital Gyrus

6	brodmann area 28

5	Hippocampus_L (aal)

5	Hippocampus

4	brodmann area 20

3	Midbrain

3	Inferior Occipital Gyrus

3	Left Brainstem

3	brodmann area 35

1	brodmann area 34

1	Cerebelum_3_L (aal)

1	brodmann area 27

----------------------

Cluster 8

Number of voxels: 52

Peak MNI coordinate: 33 39 -12

Peak MNI coordinate region: // Right Cerebrum // Frontal Lobe // Middle Frontal Gyrus // White Matter // undefined // Frontal_Mid_Orb_R (aal)

Peak intensity: 5.1118

\# voxels	structure

52	--TOTAL # VOXELS--

51	Right Cerebrum

48	Frontal Lobe

29	Frontal_Inf_Orb_R (aal)

28	Middle Frontal Gyrus

20	White Matter

20	Frontal_Mid_Orb_R (aal)

14	Gray Matter

13	Inferior Frontal Gyrus

11	brodmann area 11

3	brodmann area 47

2	Superior Frontal Gyrus

1	Frontal_Sup_Orb_R (aal)

----------------------

Cluster 9

Number of voxels: 24

Peak MNI coordinate: 60 -33 -3

Peak MNI coordinate region: // Right Cerebrum // Temporal Lobe // Middle Temporal Gyrus // White Matter // undefined // Temporal_Mid_R (aal)

Peak intensity: 3.8488

\# voxels	structure

24	--TOTAL # VOXELS--

24	Middle Temporal Gyrus

24	Right Cerebrum

24	Temporal Lobe

24	Temporal_Mid_R (aal)

23	White Matter

1	brodmann area 21

1	Gray Matter

----------------------

Cluster 10

Number of voxels: 6

Peak MNI coordinate: 0 -27 15

Peak MNI coordinate region: // Inter-Hemispheric // undefined // undefined // undefined // undefined // undefined

Peak intensity: 5.7252

\# voxels	structure

6	--TOTAL # VOXELS--

6	Inter-Hemispheric

4	White Matter

4	Corpus Callosum

----------------------

Cluster 11

Number of voxels: 561

Peak MNI coordinate: -57 -12 48

Peak MNI coordinate region: // Left Cerebrum // Parietal Lobe // Postcentral Gyrus // undefined // undefined // Postcentral_L (aal)

Peak intensity: 5.3379

\# voxels	structure

561	--TOTAL # VOXELS--

535	Left Cerebrum

350	Postcentral_L (aal)

318	Frontal Lobe

272	Precentral Gyrus

240	Gray Matter

222	White Matter

217	Parietal Lobe

211	Postcentral Gyrus

147	Precentral_L (aal)

92	brodmann area 6

56	brodmann area 3

52	brodmann area 4

29	Middle Frontal Gyrus

19	Frontal_Sup_L (aal)

19	brodmann area 2

18	Parietal_Inf_L (aal)

14	Sub-Gyral

10	brodmann area 1

7	brodmann area 40

6	Superior Frontal Gyrus

5	Inferior Parietal Lobule

4	Rolandic_Oper_L (aal)

3	SupraMarginal_L (aal)

3	brodmann area 43

----------------------

Cluster 12

Number of voxels: 377

Peak MNI coordinate: 48 -60 27

Peak MNI coordinate region: // Right Cerebrum // Temporal Lobe // Superior Temporal Gyrus // White Matter // undefined // Angular_R (aal)

Peak intensity: 5.4917

\# voxels	structure

377	--TOTAL # VOXELS--

342	Right Cerebrum

252	Angular_R (aal)

194	White Matter

183	Parietal Lobe

159	Temporal Lobe

131	Gray Matter

99	brodmann area 39

85	Angular Gyrus

83	Superior Temporal Gyrus

57	Inferior Parietal Lobule

53	Temporal_Mid_R (aal)

48	Middle Temporal Gyrus

47	Supramarginal Gyrus

25	Occipital_Mid_R (aal)

16	brodmann area 40

15	Precuneus

15	Temporal_Sup_R (aal)

8	brodmann area 7

5	brodmann area 19

4	Sub-Gyral

3	Superior Parietal Lobule

3	brodmann area 22

1	Occipital_Sup_R (aal)

1	Parietal_Sup_R (aal)

----------------------

Cluster 13

Number of voxels: 465

Peak MNI coordinate: 48 -18 63

Peak MNI coordinate region: // undefined // undefined // undefined // undefined // undefined // Precentral_R (aal)

Peak intensity: 7.328

\# voxels	structure

465	--TOTAL # VOXELS--

401	Right Cerebrum

321	Frontal Lobe

289	Precentral Gyrus

211	Gray Matter

208	Precentral_R (aal)

205	Postcentral_R (aal)

103	brodmann area 6

101	White Matter

79	Postcentral Gyrus

78	Parietal Lobe

49	brodmann area 4

40	brodmann area 3

20	Middle Frontal Gyrus

12	Inferior Frontal Gyrus

11	Frontal_Mid_R (aal)

10	Frontal_Sup_R (aal)

9	brodmann area 43

6	brodmann area 9

6	Rolandic_Oper_R (aal)

2	Temporal Lobe

2	brodmann area 1

1	brodmann area 44

1	brodmann area 2

----------------------

Cluster 14

Number of voxels: 511

Peak MNI coordinate: -48 -66 27

Peak MNI coordinate region: // Left Cerebrum // Temporal Lobe // Middle Temporal Gyrus // Gray Matter // brodmann area 39 // Angular_L (aal)

Peak intensity: 6.4387

\# voxels	structure

511	--TOTAL # VOXELS--

442	Left Cerebrum

250	Parietal Lobe

234	Angular_L (aal)

231	White Matter

194	Gray Matter

184	Temporal Lobe

118	brodmann area 39

104	Middle Temporal Gyrus

102	Angular Gyrus

100	Occipital_Mid_L (aal)

78	Precuneus

49	Inferior Parietal Lobule

49	Superior Temporal Gyrus

47	Temporal_Mid_L (aal)

47	brodmann area 19

38	Parietal_Inf_L (aal)

31	Supramarginal Gyrus

19	brodmann area 7

13	Superior Parietal Lobule

12	Parietal_Sup_L (aal)

10	brodmann area 40

8	Occipital Lobe

7	Sub-Gyral

6	Superior Occipital Gyrus

1	Cuneus

1	Occipital_Sup_L (aal)

1	SupraMarginal_L (aal)

----------------------

Cluster 15

Number of voxels: 30

Peak MNI coordinate: 21 -87 45

Peak MNI coordinate region: // undefined // undefined // undefined // undefined // undefined // undefined

Peak intensity: 3.4591

\# voxels	structure

30	--TOTAL # VOXELS--

16	Occipital_Sup_R (aal)

13	Right Cerebrum

11	Parietal Lobe

11	Precuneus

9	Gray Matter

7	brodmann area 19

5	Cuneus_R (aal)

5	Occipital_Mid_R (aal)

2	Occipital Lobe

2	brodmann area 7

2	Cuneus

1	White Matter

----------------------

Cluster 16

Number of voxels: 1

Peak MNI coordinate: 15 -24 45

Peak MNI coordinate region: // Right Cerebrum // Frontal Lobe // Sub-Gyral // White Matter // undefined // Cingulum_Mid_R (aal)

Peak intensity: 2.6674

\# voxels	structure

1	--TOTAL # VOXELS--

1	Cingulum_Mid_R (aal)

1	Frontal Lobe

1	Right Cerebrum

1	Sub-Gyral

1	White Matter

>>

This report is based on CUI Xu's xjview. (http://www.alivelearn.net/xjview/)

Revised by YAN Chao-Gan and ZHU Wei-Xuan 20091108: suitable for different Cluster Connectivity Criterion: surface connected, edge connected, corner connected.

Number of clusters found: 22

----------------------

Cluster 1

Number of voxels: 25

Peak MNI coordinate: 27 -84 -51

Peak MNI coordinate region: // undefined // undefined // undefined // undefined // undefined // undefined

Peak intensity: -3.6361

\# voxels	structure

25	--TOTAL # VOXELS--

9	Inferior Semi-Lunar Lobule

9	Right Cerebellum

9	Cerebellum Posterior Lobe

3	Cerebelum_Crus2_R (aal)

3	Cerebelum_7b_R (aal)

1	Cerebelum_8_R (aal)

----------------------

Cluster 2

Number of voxels: 990

Peak MNI coordinate: -30 -57 -36

Peak MNI coordinate region: // Left Cerebellum // Cerebellum Anterior Lobe // undefined // undefined // undefined // Cerebelum_6_L (aal)

Peak intensity: -7.527

\# voxels	structure

990	--TOTAL # VOXELS--

830	Left Cerebellum

746	Cerebellum Posterior Lobe

256	Cerebelum_Crus1_L (aal)

228	Cerebelum_Crus2_L (aal)

211	Cerebellar Tonsil

189	Declive

159	Inferior Semi-Lunar Lobule

114	Cerebelum_8_L (aal)

99	Pyramis

94	Cerebellum Anterior Lobe

77	Cerebelum_7b_L (aal)

69	Cerebelum_6_L (aal)

55	Culmen

50	Tuber

27	Uvula

23	Right Cerebellum

10	Dentate

9	Cerebelum_Crus2_R (aal)

8	Vermis_7 (aal)

7	Cerebelum_10_L (aal)

6	Declive of Vermis

5	Left Cerebrum

5	Temporal Lobe

5	Fusiform Gyrus

4	Cerebelum_7b_R (aal)

3	Cerebelum_4_5_L (aal)

3	Vermis_8 (aal)

3	brodmann area 37

3	Gray Matter

2	Tuber of Vermis

2	White Matter

1	Uvula of Vermis

1	Temporal_Inf_L (aal)

1	Pyramis of Vermis

----------------------

Cluster 3

Number of voxels: 47

Peak MNI coordinate: 39 -48 -54

Peak MNI coordinate region: // undefined // undefined // undefined // undefined // undefined // Cerebelum_8_R (aal)

Peak intensity: -4.6276

\# voxels	structure

47	--TOTAL # VOXELS--

44	Cerebelum_8_R (aal)

28	Cerebellar Tonsil

28	Right Cerebellum

28	Cerebellum Posterior Lobe

2	Cerebelum_7b_R (aal)

1	Cerebelum_9_R (aal)

----------------------

Cluster 4

Number of voxels: 45

Peak MNI coordinate: -9 -18 -45

Peak MNI coordinate region: // undefined // undefined // undefined // undefined // undefined // undefined

Peak intensity: -3.525

\# voxels	structure

45	--TOTAL # VOXELS--

19	Pons

19	Left Brainstem

2	Cerebelum_10_L (aal)

----------------------

Cluster 5

Number of voxels: 165

Peak MNI coordinate: -51 -27 -30

Peak MNI coordinate region: // undefined // undefined // undefined // undefined // undefined // undefined

Peak intensity: -5.8515

\# voxels	structure

165	--TOTAL # VOXELS--

72	Left Cerebrum

70	Temporal Lobe

64	Temporal_Inf_L (aal)

42	Gray Matter

35	Inferior Temporal Gyrus

29	Fusiform Gyrus

28	brodmann area 20

20	White Matter

13	brodmann area 37

4	Sub-Gyral

2	Parahippocampa Gyrus

2	Fusiform_L (aal)

2	Limbic Lobe

2	Middle Temporal Gyrus

1	Cerebelum_6_L (aal)

1	brodmann area 36

----------------------

Cluster 6

Number of voxels: 69

Peak MNI coordinate: 15 -48 -24

Peak MNI coordinate region: // Right Cerebellum // Cerebellum Anterior Lobe // Culmen // undefined // undefined // Cerebelum_4_5_R (aal)

Peak intensity: -3.7696

\# voxels	structure

69	--TOTAL # VOXELS--

68	Right Cerebellum

52	Cerebellum Anterior Lobe

29	Culmen

19	Cerebelum_6_R (aal)

16	Cerebellum Posterior Lobe

15	Dentate

11	Cerebelum_Crus1_R (aal)

8	Cerebelum_4_5_R (aal)

6	Declive

6	Pyramis

4	Uvula

1	Right Brainstem

1	Pons

----------------------

Cluster 7

Number of voxels: 73

Peak MNI coordinate: 63 -45 -24

Peak MNI coordinate region: // undefined // undefined // undefined // undefined // undefined // Temporal_Inf_R (aal)

Peak intensity: -3.596

\# voxels	structure

73	--TOTAL # VOXELS--

57	Temporal_Inf_R (aal)

41	Right Cerebrum

41	Temporal Lobe

19	Fusiform Gyrus

19	Inferior Temporal Gyrus

18	Gray Matter

14	White Matter

9	brodmann area 37

9	brodmann area 20

2	Sub-Gyral

1	Middle Temporal Gyrus

----------------------

Cluster 8

Number of voxels: 627

Peak MNI coordinate: 36 45 -3

Peak MNI coordinate region: // Right Cerebrum // Frontal Lobe // Middle Frontal Gyrus // White Matter // undefined // Frontal_Mid_Orb_R (aal)

Peak intensity: -7.7673

\# voxels	structure

627	--TOTAL # VOXELS--

384	Right Cerebrum

368	Frontal Lobe

195	Middle Frontal Gyrus

192	Gray Matter

184	White Matter

159	Frontal_Mid_R (aal)

99	Frontal_Mid_Orb_R (aal)

75	Inferior Frontal Gyrus

75	Superior Frontal Gyrus

66	brodmann area 10

59	Left Cerebrum

51	Frontal_Inf_Orb_R (aal)

50	Limbic Lobe

48	brodmann area 11

38	Frontal_Sup_Orb_R (aal)

36	Uncus

35	ParaHippocampal_L (aal)

24	brodmann area 47

20	Temporal Lobe

20	Temporal_Pole_Sup_L (aal)

18	ParaHippocampal_R (aal)

17	Superior Temporal Gyrus

14	Parahippocampa Gyrus

14	brodmann area 28

13	Sub-Gyral

13	brodmann area 46

10	brodmann area 34

7	Frontal_Inf_Tri_R (aal)

6	Orbital Gyrus

5	brodmann area 9

4	Amygdala_L (aal)

4	brodmann area 38

3	Temporal_Pole_Sup_R (aal)

3	Amygdala

3	Olfactory_R (aal)

3	Left Brainstem

2	Frontal_Inf_Orb_L (aal)

2	Hippocampus_L (aal)

2	Midbrain

1	brodmann area 25

1	Frontal_Sup_R (aal)

1	Pons

1	Subcallosal Gyrus

----------------------

Cluster 9

Number of voxels: 124

Peak MNI coordinate: -24 39 -21

Peak MNI coordinate region: // Left Cerebrum // Frontal Lobe // Superior Frontal Gyrus // undefined // undefined // undefined

Peak intensity: -6.3937

\# voxels	structure

124	--TOTAL # VOXELS--

115	Left Cerebrum

115	Frontal Lobe

60	Gray Matter

48	brodmann area 11

42	White Matter

33	Frontal_Mid_Orb_L (aal)

32	Inferior Frontal Gyrus

29	Superior Frontal Gyrus

26	Middle Frontal Gyrus

22	Frontal_Sup_Orb_L (aal)

21	Frontal_Inf_Orb_L (aal)

16	Orbital Gyrus

12	brodmann area 47

6	Sub-Gyral

6	Rectal Gyrus

----------------------

Cluster 10

Number of voxels: 25

Peak MNI coordinate: 6 -21 -27

Peak MNI coordinate region: // Right Brainstem // Pons // undefined // undefined // undefined // undefined

Peak intensity: -3.4184

\# voxels	structure

25	--TOTAL # VOXELS--

25	Right Brainstem

25	Pons

----------------------

Cluster 11

Number of voxels: 391

Peak MNI coordinate: 39 -3 -18

Peak MNI coordinate region: // Right Cerebrum // Temporal Lobe // Sub-Gyral // White Matter // undefined // undefined

Peak intensity: -7.7994

\# voxels	structure

391	--TOTAL # VOXELS--

389	Right Cerebrum

243	Temporal Lobe

193	Superior Temporal Gyrus

159	White Matter

138	Gray Matter

122	Temporal_Pole_Sup_R (aal)

120	Frontal Lobe

114	Inferior Frontal Gyrus

63	Frontal_Inf_Oper_R (aal)

58	brodmann area 38

43	Sub-Gyral

39	Temporal_Sup_R (aal)

33	Frontal_Inf_Orb_R (aal)

24	Frontal_Inf_Tri_R (aal)

24	brodmann area 22

23	brodmann area 47

23	Insula_R (aal)

18	brodmann area 45

16	Frontal-Temporal Space

14	brodmann area 44

12	Rolandic_Oper_R (aal)

9	Precentral Gyrus

3	Sub-lobar

3	Heschl_R (aal)

2	Extra-Nuclear

1	Insula

1	brodmann area 13

----------------------

Cluster 12

Number of voxels: 103

Peak MNI coordinate: 60 -57 -15

Peak MNI coordinate region: // Right Cerebrum // Temporal Lobe // Inferior Temporal Gyrus // undefined // undefined // Temporal_Inf_R (aal)

Peak intensity: -4.6957

\# voxels	structure

103	--TOTAL # VOXELS--

91	Right Cerebrum

78	Temporal_Inf_R (aal)

76	Temporal Lobe

41	Gray Matter

40	Inferior Temporal Gyrus

38	White Matter

23	brodmann area 37

15	Middle Temporal Gyrus

15	Occipital Lobe

14	Middle Occipital Gyrus

12	brodmann area 20

11	Sub-Gyral

8	Fusiform Gyrus

5	brodmann area 19

4	Temporal_Mid_R (aal)

1	Cerebelum_Crus1_R (aal)

1	brodmann area 21

1	Occipital_Inf_R (aal)

1	Fusiform_R (aal)

----------------------

Cluster 13

Number of voxels: 13

Peak MNI coordinate: -42 -9 -15

Peak MNI coordinate region: // Left Cerebrum // Temporal Lobe // Sub-Gyral // White Matter // undefined // undefined

Peak intensity: -6.3094

\# voxels	structure

13	--TOTAL # VOXELS--

13	Sub-Gyral

13	Temporal Lobe

13	White Matter

13	Left Cerebrum

1	Temporal_Mid_L (aal)

----------------------

Cluster 14

Number of voxels: 42

Peak MNI coordinate: -24 -102 -15

Peak MNI coordinate region: // undefined // undefined // undefined // undefined // undefined // undefined

Peak intensity: -4.9

\# voxels	structure

42	--TOTAL # VOXELS--

29	Occipital Lobe

29	Left Cerebrum

16	Gray Matter

15	brodmann area 18

15	Inferior Occipital Gyrus

12	Lingual Gyrus

5	Lingual_L (aal)

4	White Matter

1	brodmann area 17

----------------------

Cluster 15

Number of voxels: 115

Peak MNI coordinate: -60 -63 -6

Peak MNI coordinate region: // Left Cerebrum // Temporal Lobe // Middle Temporal Gyrus // Gray Matter // brodmann area 37 // undefined

Peak intensity: -5.5316

\# voxels	structure

115	--TOTAL # VOXELS--

108	Left Cerebrum

63	Temporal Lobe

61	Gray Matter

45	Occipital Lobe

40	Middle Temporal Gyrus

40	Middle Occipital Gyrus

38	White Matter

30	brodmann area 37

30	Temporal_Inf_L (aal)

25	Inferior Temporal Gyrus

22	Occipital_Inf_L (aal)

19	Temporal_Mid_L (aal)

18	brodmann area 19

5	Fusiform Gyrus

4	brodmann area 20

2	brodmann area 39

2	brodmann area 21

1	Occipital_Mid_L (aal)

----------------------

Cluster 16

Number of voxels: 921

Peak MNI coordinate: -39 -42 33

Peak MNI coordinate region: // Left Cerebrum // Parietal Lobe // Supramarginal Gyrus // White Matter // undefined // undefined

Peak intensity: -7.8752

\# voxels	structure

921	--TOTAL # VOXELS--

888	Left Cerebrum

576	Parietal Lobe

398	White Matter

395	Gray Matter

357	Inferior Parietal Lobule

248	brodmann area 40

243	Parietal_Inf_L (aal)

239	Temporal Lobe

221	Superior Temporal Gyrus

199	SupraMarginal_L (aal)

188	Temporal_Sup_L (aal)

96	Supramarginal Gyrus

65	Sub-Gyral

64	Postcentral Gyrus

56	Temporal_Pole_Sup_L (aal)

50	Frontal Lobe

48	Frontal_Inf_Oper_L (aal)

36	brodmann area 22

33	Inferior Frontal Gyrus

32	brodmann area 42

22	brodmann area 38

20	brodmann area 2

19	Frontal-Temporal Space

16	Postcentral_L (aal)

16	brodmann area 44

16	Precentral Gyrus

14	Angular_L (aal)

10	Rolandic_Oper_L (aal)

7	Transverse Temporal Gyrus

6	Precentral_L (aal)

6	Temporal_Mid_L (aal)

6	brodmann area 41

5	Middle Temporal Gyrus

4	Frontal_Inf_Tri_L (aal)

4	Insula

4	Sub-lobar

4	brodmann area 1

3	brodmann area 21

3	brodmann area 43

2	brodmann area 9

1	brodmann area 47

----------------------

Cluster 17

Number of voxels: 383

Peak MNI coordinate: -48 48	6

Peak MNI coordinate region: // Left Cerebrum // Frontal Lobe // Inferior Frontal Gyrus // White Matter // undefined // Frontal_Inf_Tri_L (aal)

Peak intensity: -7.0274

\# voxels	structure

383	--TOTAL # VOXELS--

376	Frontal Lobe

376	Left Cerebrum

232	White Matter

222	Middle Frontal Gyrus

193	Frontal_Mid_L (aal)

139	Frontal_Inf_Tri_L (aal)

127	Gray Matter

119	Inferior Frontal Gyrus

60	brodmann area 10

45	brodmann area 46

17	Sub-Gyral

17	Superior Frontal Gyrus

17	Frontal_Inf_Orb_L (aal)

9	Frontal_Mid_Orb_L (aal)

7	brodmann area 47

4	brodmann area 9

4	brodmann area 45

----------------------

Cluster 18

Number of voxels: 5

Peak MNI coordinate: -36 -51	9

Peak MNI coordinate region: // Left Cerebrum // Temporal Lobe // Sub-Gyral // White Matter // undefined // undefined

Peak intensity: -3.8448

\# voxels	structure

5	--TOTAL # VOXELS--

5	Temporal Lobe

5	White Matter

5	Left Cerebrum

4	Sub-Gyral

1	Superior Temporal Gyrus

----------------------

Cluster 19

Number of voxels: 1053

Peak MNI coordinate: -3 12 21

Peak MNI coordinate region: // Left Cerebrum // Sub-lobar // Extra-Nuclear // White Matter // Corpus Callosum // undefined

Peak intensity: -9.9441

\# voxels	structure

1053	--TOTAL # VOXELS--

582	Right Cerebrum

575	Limbic Lobe

483	White Matter

432	Cingulate Gyrus

373	Gray Matter

348	Left Cerebrum

232	Frontal Lobe

200	Cingulum_Mid_R (aal)

153	Anterior Cingulate

142	Cingulum_Mid_L (aal)

132	brodmann area 24

129	Cingulum_Ant_L (aal)

123	Inter-Hemispheric

117	Corpus Callosum

110	brodmann area 32

102	Cingulum_Ant_R (aal)

94	Sub-lobar

90	Extra-Nuclear

71	Sub-Gyral

65	Medial Frontal Gyrus

58	Frontal_Sup_Medial_L (aal)

56	Middle Frontal Gyrus

51	Frontal_Sup_R (aal)

47	Frontal_Mid_R (aal)

39	brodmann area 6

38	brodmann area 31

30	Supp_Motor_Area_R (aal)

29	Parietal Lobe

20	brodmann area 9

16	Frontal_Sup_Medial_R (aal)

16	brodmann area 23

14	Superior Frontal Gyrus

14	Posterior Cingulate

13	Precuneus

11	Cerebro-Spinal Fluid

11	Lateral Ventricle

10	Precentral_R (aal)

10	Precuneus_R (aal)

10	brodmann area 33

9	Supp_Motor_Area_L (aal)

7	Paracentral Lobule

4	Paracentral_Lobule_R (aal)

4	brodmann area 7

3	Cingulum_Post_R (aal)

3	brodmann area 8

2	Cingulum_Post_L (aal)

1	brodmann area 5

1	Precuneus_L (aal)

1	Frontal_Sup_L (aal)

----------------------

Cluster 20

Number of voxels: 11

Peak MNI coordinate: 0 -9 9

Peak MNI coordinate region: // Inter-Hemispheric // undefined // undefined // undefined // undefined // undefined

Peak intensity: -4.2538

\# voxels	structure

11	--TOTAL # VOXELS--

6	Inter-Hemispheric

5	Medial Dorsal Nucleus

5	Sub-lobar

5	Thalamus

5	Gray Matter

3	Thalamus_L (aal)

3	Left Cerebrum

2	Thalamus_R (aal)

2	Right Cerebrum

----------------------

Cluster 21

Number of voxels: 1187

Peak MNI coordinate: 36 -45 33

Peak MNI coordinate region: // Right Cerebrum // Parietal Lobe // Sub-Gyral // White Matter // undefined // undefined

Peak intensity: -9.8071

\# voxels	structure

1187	--TOTAL # VOXELS--

1050	Right Cerebrum

1005	Parietal Lobe

550	White Matter

534	Inferior Parietal Lobule

515	SupraMarginal_R (aal)

419	Gray Matter

314	brodmann area 40

221	Parietal_Inf_R (aal)

218	Postcentral Gyrus

106	Sub-Gyral

101	Supramarginal Gyrus

79	Temporal_Sup_R (aal)

54	Parietal_Sup_R (aal)

50	Postcentral_R (aal)

50	brodmann area 2

49	Precuneus

30	Temporal Lobe

21	Superior Temporal Gyrus

20	Angular_R (aal)

17	Precuneus_R (aal)

15	Rolandic_Oper_R (aal)

13	Occipital_Sup_R (aal)

13	Frontal Lobe

11	brodmann area 42

11	brodmann area 7

11	brodmann area 1

9	brodmann area 3

7	Superior Parietal Lobule

5	Precentral Gyrus

4	brodmann area 41

3	Paracentral Lobule

3	Insula

3	Cuneus_R (aal)

3	brodmann area 43

3	Transverse Temporal Gyrus

3	brodmann area 4

2	brodmann area 5

2	Occipital_Mid_R (aal)

2	Sub-lobar

1	brodmann area 13

----------------------

Cluster 22

Number of voxels: 20

Peak MNI coordinate: -21 -30 51

Peak MNI coordinate region: // Left Cerebrum // Parietal Lobe // Postcentral Gyrus // Gray Matter // brodmann area 3 // undefined

Peak intensity: -4.3959

\# voxels	structure

20	--TOTAL # VOXELS--

20	Left Cerebrum

14	White Matter

13	Sub-Gyral

12	Frontal Lobe

8	Parietal Lobe

6	Gray Matter

4	Postcentral Gyrus

3	brodmann area 3

3	brodmann area 4

3	Precentral Gyrus

>>
