# Supplementary material for: Abnormal coordination of executive control network and default mode network in cognitive impairment after deep nuclei intracerebral hemorrhage
Source: Front Neurol. 2026 Sep 16;17:1915567. doi: 10.3389/fneur.2026.1915567 (PMC13623672; doi:10.3389/fneur.2026.1915567)
Supplement: Supplementary file 5 [file Table_3.docx]

Supplementary Table 3 **Compared with HC, brain detail regions of DMN with PCC as ROI**

This report is based on CUI Xu's xjview. (http://www.alivelearn.net/xjview/)

Revised by YAN Chao-Gan and ZHU Wei-Xuan 20091108: suitable for different Cluster Connectivity Criterion: surface connected, edge connected, corner connected.

Number of clusters found: 6

----------------------

Cluster 1

Number of voxels: 31

Peak MNI coordinate: -3 -18 -42

Peak MNI coordinate region: // Left Brainstem // Pons // undefined // undefined // undefined // undefined

Peak intensity: -3.9009

\# voxels	structure

31	--TOTAL # VOXELS--

21	Pons

21	Left Brainstem

8	Right Brainstem

8	Medulla

----------------------

Cluster 2

Number of voxels: 890

Peak MNI coordinate: 63	0 -15

Peak MNI coordinate region: // Right Cerebrum // Temporal Lobe // Middle Temporal Gyrus // Gray Matter // brodmann area 21 // Temporal_Mid_R (aal)

Peak intensity: -6.1164

\# voxels	structure

890	--TOTAL # VOXELS--

755	Right Cerebrum

549	Temporal Lobe

333	Gray Matter

295	Superior Temporal Gyrus

292	White Matter

231	Temporal_Sup_R (aal)

185	Middle Temporal Gyrus

166	Temporal_Mid_R (aal)

147	Frontal Lobe

136	Temporal_Pole_Sup_R (aal)

135	Precentral Gyrus

130	Postcentral_R (aal)

102	brodmann area 21

60	brodmann area 38

52	Temporal_Pole_Mid_R (aal)

50	brodmann area 22

49	Rolandic_Oper_R (aal)

45	Parietal Lobe

42	Postcentral Gyrus

32	brodmann area 6

27	Precentral_R (aal)

26	Transverse Temporal Gyrus

25	Temporal_Inf_R (aal)

22	Inferior Temporal Gyrus

19	Inferior Frontal Gyrus

19	brodmann area 43

18	brodmann area 42

17	brodmann area 4

14	Sub-Gyral

14	Heschl_R (aal)

8	brodmann area 20

8	brodmann area 41

8	Frontal_Inf_Orb_R (aal)

7	brodmann area 47

6	SupraMarginal_R (aal)

6	Limbic Lobe

5	Insula_R (aal)

5	Uncus

4	Insula

4	brodmann area 3

3	Sub-lobar

3	Frontal-Temporal Space

3	ParaHippocampal_R (aal)

2	brodmann area 28

2	Fusiform Gyrus

2	brodmann area 40

1	brodmann area 13

1	Parahippocampa Gyrus

1	brodmann area 1

----------------------

Cluster 3

Number of voxels: 5826

Peak MNI coordinate: 6 39 -18

Peak MNI coordinate region: // Right Cerebrum // Frontal Lobe // Medial Frontal Gyrus // White Matter // undefined // Rectus_R (aal)

Peak intensity: -8.369

\# voxels	structure

5826	--TOTAL # VOXELS--

4045	Left Cerebrum

2574	White Matter

2125	Gray Matter

1462	Frontal Lobe

1196	Occipital Lobe

1052	Right Cerebrum

964	Temporal Lobe

856	Limbic Lobe

513	Postcentral_L (aal)

465	Medial Frontal Gyrus

460	Precentral Gyrus

432	Parietal Lobe

425	Lingual Gyrus

408	Superior Temporal Gyrus

379	Temporal_Mid_L (aal)

366	Calcarine_L (aal)

313	Postcentral Gyrus

303	Middle Temporal Gyrus

275	Temporal_Sup_L (aal)

275	Superior Frontal Gyrus

275	Cuneus

274	Inter-Hemispheric

268	Lingual_L (aal)

256	Parahippocampa Gyrus

237	Precentral_L (aal)

235	Cingulate Gyrus

220	Sub-Gyral

218	brodmann area 18

214	brodmann area 10

207	brodmann area 11

193	Fusiform_L (aal)

190	Anterior Cingulate

184	Sub-lobar

180	Rectus_L (aal)

169	Inferior Occipital Gyrus

163	Extra-Nuclear

160	Lingual_R (aal)

156	Frontal_Med_Orb_L (aal)

154	Middle Occipital Gyrus

153	Frontal_Med_Orb_R (aal)

149	Precuneus

145	Cingulum_Ant_L (aal)

144	Corpus Callosum

144	Calcarine_R (aal)

137	Rectus_R (aal)

136	Frontal_Sup_Medial_L (aal)

132	Occipital_Inf_L (aal)

131	brodmann area 21

131	ParaHippocampal_L (aal)

128	Left Cerebellum

124	Posterior Cingulate

118	Cerebellum Anterior Lobe

118	Culmen

114	Occipital_Mid_L (aal)

112	brodmann area 24

109	brodmann area 17

108	brodmann area 4

105	brodmann area 19

104	Frontal_Sup_Medial_R (aal)

103	Fusiform Gyrus

100	Frontal_Sup_L (aal)

94	brodmann area 6

93	Cingulum_Mid_L (aal)

92	Temporal_Pole_Sup_L (aal)

83	brodmann area 3

83	brodmann area 22

76	Cingulum_Ant_R (aal)

75	Cingulum_Mid_R (aal)

74	Cuneus_L (aal)

71	brodmann area 30

67	Rectal Gyrus

63	Inferior Temporal Gyrus

58	brodmann area 32

53	brodmann area 31

50	Temporal_Pole_Mid_L (aal)

48	brodmann area 38

47	Frontal_Sup_R (aal)

46	Transverse Temporal Gyrus

44	Orbital Gyrus

42	Temporal_Inf_L (aal)

41	Inferior Frontal Gyrus

40	Cerebelum_6_L (aal)

39	Cerebelum_4_5_L (aal)

38	Cingulum_Post_L (aal)

37	Precuneus_L (aal)

37	brodmann area 42

36	brodmann area 37

35	brodmann area 43

34	Middle Frontal Gyrus

33	Frontal_Sup_Orb_R (aal)

33	Rolandic_Oper_L (aal)

33	Frontal_Sup_Orb_L (aal)

32	Uncus

30	Cerebelum_Crus1_L (aal)

29	SupraMarginal_L (aal)

29	brodmann area 20

26	Occipital_Sup_L (aal)

25	Hippocampus_L (aal)

25	brodmann area 41

25	brodmann area 35

24	Cerebellum Posterior Lobe

24	Declive

24	Parietal_Inf_L (aal)

23	brodmann area 36

22	brodmann area 9

22	Precuneus_R (aal)

21	Occipital_Inf_R (aal)

20	Lateral Ventricle

20	brodmann area 2

20	Cerebro-Spinal Fluid

18	brodmann area 23

16	brodmann area 7

16	Paracentral Lobule

16	brodmann area 40

14	brodmann area 28

14	Right Cerebellum

13	Paracentral_Lobule_L (aal)

13	brodmann area 25

11	Amygdala_L (aal)

10	Cuneus_R (aal)

10	Insula

10	brodmann area 34

10	brodmann area 1

9	brodmann area 39

9	brodmann area 33

8	Inferior Parietal Lobule

7	Amygdala

6	Cingulum_Post_R (aal)

6	Frontal_Inf_Oper_L (aal)

6	Cerebelum_4_5_R (aal)

5	Heschl_L (aal)

5	ParaHippocampal_R (aal)

5	Vermis_4_5 (aal)

5	brodmann area 29

4	Subcallosal Gyrus

4	brodmann area 13

4	Olfactory_L (aal)

4	brodmann area 44

3	Frontal_Inf_Orb_L (aal)

3	brodmann area 8

2	Hippocampus

2	Left Brainstem

2	brodmann area 47

2	Cerebelum_6_R (aal)

1	Midbrain

1	Supp_Motor_Area_L (aal)

1	Optic Tract

1	Pons

----------------------

Cluster 4

Number of voxels: 64

Peak MNI coordinate: 30 -15 -21

Peak MNI coordinate region: // Right Cerebrum // Limbic Lobe // Parahippocampa Gyrus // Gray Matter // Hippocampus // Hippocampus_R (aal)

Peak intensity: -5.9566

\# voxels	structure

64	--TOTAL # VOXELS--

63	Limbic Lobe

63	Parahippocampa Gyrus

63	Right Cerebrum

48	ParaHippocampal_R (aal)

44	White Matter

17	Gray Matter

10	Hippocampus_R (aal)

6	Hippocampus

5	brodmann area 35

4	brodmann area 28

2	brodmann area 36

1	Fusiform_R (aal)

----------------------

Cluster 5

Number of voxels: 1

Peak MNI coordinate: -3 -30 21

Peak MNI coordinate region: // Left Cerebrum // Limbic Lobe // Posterior Cingulate // Gray Matter // brodmann area 23 // undefined

Peak intensity: -2.6898

\# voxels	structure

1	--TOTAL # VOXELS--

1	Gray Matter

1	Left Cerebrum

1	Limbic Lobe

1	Posterior Cingulate

1	brodmann area 23

----------------------

Cluster 6

Number of voxels: 57

Peak MNI coordinate: 24 -24 51

Peak MNI coordinate region: // Right Cerebrum // Frontal Lobe // Precentral Gyrus // White Matter // undefined // undefined

Peak intensity: -3.9186

\# voxels	structure

57	--TOTAL # VOXELS--

56	Right Cerebrum

49	Frontal Lobe

47	White Matter

37	Precentral Gyrus

29	Precentral_R (aal)

14	Sub-Gyral

9	Gray Matter

7	Parietal Lobe

5	Postcentral Gyrus

5	brodmann area 4

5	Postcentral_R (aal)

3	brodmann area 6

1	brodmann area 3

>>

This report is based on CUI Xu's xjview. (http://www.alivelearn.net/xjview/)

Revised by YAN Chao-Gan and ZHU Wei-Xuan 20091108: suitable for different Cluster Connectivity Criterion: surface connected, edge connected, corner connected.

Number of clusters found: 15

----------------------

Cluster 1

Number of voxels: 2109

Peak MNI coordinate: -9 -36 -57

Peak MNI coordinate region: // undefined // undefined // undefined // undefined // undefined // undefined

Peak intensity: 6.661

\# voxels	structure

2109	--TOTAL # VOXELS--

1068	Cerebellum Posterior Lobe

812	Right Cerebellum

581	Left Cerebellum

311	Cerebellum Anterior Lobe

287	Declive

276	Inferior Semi-Lunar Lobule

204	Cerebellar Tonsil

197	Cerebelum_8_R (aal)

194	Culmen

168	Cerebelum_8_L (aal)

155	Cerebelum_Crus1_R (aal)

141	Pyramis

140	Cerebelum_Crus2_R (aal)

114	Cerebelum_Crus2_L (aal)

106	Vermis_4_5 (aal)

97	Uvula

61	Cerebelum_7b_L (aal)

58	Right Cerebrum

58	Cerebelum_Crus1_L (aal)

57	Occipital Lobe

55	Tuber

50	Cerebelum_9_R (aal)

47	Lingual Gyrus

46	Right Brainstem

45	Cerebelum_4_5_L (aal)

42	Medulla

41	Cerebelum_7b_R (aal)

40	Vermis_8 (aal)

38	Cerebelum_6_R (aal)

33	Vermis_6 (aal)

31	Left Brainstem

29	Vermis_7 (aal)

29	Cerebelum_6_L (aal)

29	Gray Matter

28	Cerebelum_4_5_R (aal)

28	Nodule

24	Cerebelum_9_L (aal)

22	Midbrain

22	Dentate

21	Vermis_9 (aal)

19	brodmann area 18

19	White Matter

16	Cerebellar Lingual

13	Pons

11	Declive of Vermis

10	Fastigium

9	Vermis_3 (aal)

9	brodmann area 17

8	Inter-Hemispheric

5	Culmen of Vermis

5	Cerebelum_3_R (aal)

4	Cerebro-Spinal Fluid

4	Vermis_10 (aal)

4	Sub-lobar

4	Fourth Ventricle

3	Cuneus

2	Uvula of Vermis

2	Tuber of Vermis

2	Fusiform Gyrus

1	Cerebelum_3_L (aal)

1	Inferior Occipital Gyrus

1	Limbic Lobe

1	Posterior Cingulate

----------------------

Cluster 2

Number of voxels: 46

Peak MNI coordinate: 57 -54 -33

Peak MNI coordinate region: // Right Cerebellum // Cerebellum Posterior Lobe // Tuber // undefined // undefined // Cerebelum_Crus1_R (aal)

Peak intensity: 4.4284

\# voxels	structure

46	--TOTAL # VOXELS--

41	Right Cerebellum

39	Cerebelum_Crus1_R (aal)

29	Cerebellum Posterior Lobe

21	Tuber

12	Culmen

12	Cerebellum Anterior Lobe

7	Cerebelum_Crus2_R (aal)

5	Cerebellar Tonsil

3	Declive

----------------------

Cluster 3

Number of voxels: 1440

Peak MNI coordinate: 33 -45	3

Peak MNI coordinate region: // Right Cerebrum // Temporal Lobe // Sub-Gyral // White Matter // undefined // undefined

Peak intensity: 10.1731

\# voxels	structure

1440	--TOTAL # VOXELS--

1161	Right Cerebrum

755	White Matter

753	Parietal Lobe

377	Gray Matter

368	Inferior Parietal Lobule

278	Temporal Lobe

248	Sub-Gyral

242	SupraMarginal_R (aal)

207	brodmann area 40

200	Parietal_Inf_R (aal)

196	Precuneus_R (aal)

195	Precuneus

147	Occipital Lobe

131	brodmann area 7

122	Parietal_Sup_R (aal)

87	Precuneus_L (aal)

78	Middle Temporal Gyrus

71	Postcentral Gyrus

69	Supramarginal Gyrus

64	Left Cerebrum

52	Occipital_Mid_R (aal)

47	Cuneus

46	Middle Occipital Gyrus

43	Inter-Hemispheric

41	Superior Parietal Lobule

35	Calcarine_R (aal)

30	Sub-lobar

28	Occipital_Sup_R (aal)

26	Angular_R (aal)

24	Fusiform_R (aal)

18	Cerebro-Spinal Fluid

18	Lateral Ventricle

13	brodmann area 18

13	Temporal_Inf_R (aal)

12	Limbic Lobe

12	Extra-Nuclear

10	Cuneus_R (aal)

9	ParaHippocampal_R (aal)

9	Parahippocampa Gyrus

8	Lingual_R (aal)

7	Temporal_Mid_R (aal)

6	Lingual Gyrus

6	brodmann area 5

5	Corpus Callosum

5	brodmann area 39

5	Superior Temporal Gyrus

4	brodmann area 2

4	Postcentral_R (aal)

4	Hippocampus_R (aal)

3	brodmann area 31

3	brodmann area 37

3	Inferior Temporal Gyrus

3	Superior Occipital Gyrus

3	Angular Gyrus

3	brodmann area 19

3	Frontal Lobe

2	Temporal_Sup_R (aal)

2	Parietal_Sup_L (aal)

2	Paracentral_Lobule_R (aal)

2	Paracentral Lobule

1	brodmann area 4

1	Paracentral_Lobule_L (aal)

1	brodmann area 36

1	Posterior Cingulate

1	Fusiform Gyrus

----------------------

Cluster 4

Number of voxels: 209

Peak MNI coordinate: 27 42 -15

Peak MNI coordinate region: // Right Cerebrum // Frontal Lobe // Middle Frontal Gyrus // undefined // undefined // Frontal_Mid_Orb_R (aal)

Peak intensity: 4.773

\# voxels	structure

209	--TOTAL # VOXELS--

209	Right Cerebrum

200	Frontal Lobe

138	Middle Frontal Gyrus

136	White Matter

109	Frontal_Mid_R (aal)

63	Gray Matter

47	Frontal_Mid_Orb_R (aal)

35	Superior Frontal Gyrus

25	brodmann area 11

24	brodmann area 10

20	Frontal_Sup_Orb_R (aal)

16	Sub-Gyral

11	Inferior Frontal Gyrus

9	brodmann area 46

7	Limbic Lobe

7	Anterior Cingulate

4	Frontal_Sup_R (aal)

2	Frontal_Inf_Orb_R (aal)

2	Sub-lobar

1	Caudate Head

1	Frontal_Inf_Tri_R (aal)

1	Lateral Ventricle

1	Caudate_R (aal)

1	Cerebro-Spinal Fluid

1	Corpus Callosum

1	brodmann area 9

1	Caudate

----------------------

Cluster 5

Number of voxels: 70

Peak MNI coordinate: -21 42 -12

Peak MNI coordinate region: // Left Cerebrum // Frontal Lobe // Middle Frontal Gyrus // Gray Matter // brodmann area 11 // Frontal_Sup_Orb_L (aal)

Peak intensity: 4.5243

\# voxels	structure

70	--TOTAL # VOXELS--

70	Frontal Lobe

70	Left Cerebrum

52	Middle Frontal Gyrus

45	White Matter

30	Frontal_Mid_Orb_L (aal)

26	Frontal_Inf_Orb_L (aal)

20	Gray Matter

19	brodmann area 11

12	Inferior Frontal Gyrus

7	Frontal_Inf_Tri_L (aal)

5	Sub-Gyral

3	Frontal_Sup_Orb_L (aal)

1	brodmann area 47

1	Superior Frontal Gyrus

----------------------

Cluster 6

Number of voxels: 51

Peak MNI coordinate: 51 15	0

Peak MNI coordinate region: // Right Cerebrum // Frontal Lobe // Inferior Frontal Gyrus // undefined // undefined // Frontal_Inf_Oper_R (aal)

Peak intensity: 5.0852

\# voxels	structure

51	--TOTAL # VOXELS--

51	Right Cerebrum

37	Frontal Lobe

32	Inferior Frontal Gyrus

27	Frontal_Inf_Oper_R (aal)

17	Gray Matter

8	White Matter

8	Frontal-Temporal Space

7	brodmann area 47

7	Rolandic_Oper_R (aal)

6	Temporal Lobe

6	Frontal_Inf_Orb_R (aal)

5	Precentral Gyrus

5	Superior Temporal Gyrus

4	brodmann area 44

4	brodmann area 45

2	Frontal_Inf_Tri_R (aal)

1	Temporal_Pole_Sup_R (aal)

1	Insula_R (aal)

1	brodmann area 22

1	brodmann area 38

----------------------

Cluster 7

Number of voxels: 224

Peak MNI coordinate: -21 -45	9

Peak MNI coordinate region: // Left Cerebrum // Sub-lobar // Lateral Ventricle // Cerebro-Spinal Fluid // undefined // undefined

Peak intensity: 10.1395

\# voxels	structure

224	--TOTAL # VOXELS--

224	Left Cerebrum

184	White Matter

112	Sub-Gyral

91	Temporal Lobe

73	Sub-lobar

37	Cerebro-Spinal Fluid

37	Lateral Ventricle

36	Extra-Nuclear

32	Occipital Lobe

22	Parietal Lobe

20	Middle Occipital Gyrus

10	Corpus Callosum

9	Precuneus_L (aal)

6	Lingual_L (aal)

5	Limbic Lobe

5	Inferior Parietal Lobule

5	Superior Temporal Gyrus

4	ParaHippocampal_L (aal)

3	Parahippocampa Gyrus

3	Fusiform_L (aal)

3	Gray Matter

2	Cuneus

2	Occipital_Mid_L (aal)

2	Calcarine_L (aal)

1	Precuneus

1	SupraMarginal_L (aal)

1	Supramarginal Gyrus

1	Middle Temporal Gyrus

1	Frontal Lobe

1	brodmann area 13

1	brodmann area 19

1	brodmann area 29

1	Posterior Cingulate

----------------------

Cluster 8

Number of voxels: 8

Peak MNI coordinate: -9 24	0

Peak MNI coordinate region: // Left Cerebrum // Sub-lobar // Lateral Ventricle // Cerebro-Spinal Fluid // undefined // Caudate_L (aal)

Peak intensity: 3.5688

\# voxels	structure

8	--TOTAL # VOXELS--

8	Left Cerebrum

6	Sub-lobar

5	Caudate_L (aal)

4	White Matter

3	Cerebro-Spinal Fluid

3	Lateral Ventricle

2	Anterior Cingulate

2	Extra-Nuclear

2	Limbic Lobe

1	Caudate

1	Olfactory_L (aal)

1	Gray Matter

1	Caudate Head

1	Corpus Callosum

----------------------

Cluster 9

Number of voxels: 2

Peak MNI coordinate: -6 -36	0

Peak MNI coordinate region: // Left Cerebrum // Sub-lobar // Extra-Nuclear // White Matter // undefined // undefined

Peak intensity: 4.2285

\# voxels	structure

2	--TOTAL # VOXELS--

2	Left Cerebrum

1	Limbic Lobe

1	Lingual_L (aal)

1	Parahippocampa Gyrus

1	Sub-lobar

1	White Matter

1	Extra-Nuclear

----------------------

Cluster 10

Number of voxels: 26

Peak MNI coordinate: 0 -27 15

Peak MNI coordinate region: // Inter-Hemispheric // undefined // undefined // undefined // undefined // undefined

Peak intensity: 7.0189

\# voxels	structure

26	--TOTAL # VOXELS--

20	White Matter

20	Corpus Callosum

16	Inter-Hemispheric

10	Extra-Nuclear

10	Sub-lobar

7	Right Cerebrum

3	Left Cerebrum

1	Cingulum_Post_L (aal)

----------------------

Cluster 11

Number of voxels: 1

Peak MNI coordinate: -6	9 15

Peak MNI coordinate region: // Left Cerebrum // Sub-lobar // Lateral Ventricle // Cerebro-Spinal Fluid // undefined // undefined

Peak intensity: 3.636

\# voxels	structure

1	--TOTAL # VOXELS--

1	Lateral Ventricle

1	Left Cerebrum

1	Sub-lobar

1	Cerebro-Spinal Fluid

----------------------

Cluster 12

Number of voxels: 62

Peak MNI coordinate: -54 -45 45

Peak MNI coordinate region: // Left Cerebrum // Parietal Lobe // Inferior Parietal Lobule // White Matter // undefined // Parietal_Inf_L (aal)

Peak intensity: 4.2263

\# voxels	structure

62	--TOTAL # VOXELS--

50	Left Cerebrum

50	Parietal Lobe

50	Inferior Parietal Lobule

41	Parietal_Inf_L (aal)

37	brodmann area 40

37	Gray Matter

9	White Matter

3	SupraMarginal_L (aal)

1	Supramarginal Gyrus

----------------------

Cluster 13

Number of voxels: 248

Peak MNI coordinate: 3 21 66

Peak MNI coordinate region: // undefined // undefined // undefined // undefined // undefined // undefined

Peak intensity: 5.5224

\# voxels	structure

248	--TOTAL # VOXELS--

85	Frontal Lobe

82	Gray Matter

80	Frontal_Sup_Medial_L (aal)

68	Inter-Hemispheric

63	Right Cerebrum

45	Frontal_Sup_Medial_R (aal)

43	Medial Frontal Gyrus

42	brodmann area 8

42	Superior Frontal Gyrus

38	Supp_Motor_Area_L (aal)

31	brodmann area 6

30	Left Cerebrum

25	Supp_Motor_Area_R (aal)

8	brodmann area 32

8	Cingulate Gyrus

6	Limbic Lobe

3	White Matter

1	brodmann area 9

1	Cingulum_Mid_R (aal)

----------------------

Cluster 14

Number of voxels: 197

Peak MNI coordinate: 21	9 66

Peak MNI coordinate region: // Right Cerebrum // Frontal Lobe // Middle Frontal Gyrus // Gray Matter // brodmann area 6 // Frontal_Sup_R (aal)

Peak intensity: 4.4946

\# voxels	structure

197	--TOTAL # VOXELS--

145	Right Cerebrum

145	Frontal Lobe

131	Frontal_Sup_R (aal)

94	Superior Frontal Gyrus

69	Gray Matter

57	White Matter

53	brodmann area 6

49	Middle Frontal Gyrus

19	Frontal_Mid_R (aal)

16	brodmann area 8

13	Supp_Motor_Area_R (aal)

2	Sub-Gyral

----------------------

Cluster 15

Number of voxels: 22

Peak MNI coordinate: -18 18 66

Peak MNI coordinate region: // Left Cerebrum // Frontal Lobe // Superior Frontal Gyrus // Gray Matter // brodmann area 6 // Frontal_Sup_L (aal)

Peak intensity: 3.5593

\# voxels	structure

22	--TOTAL # VOXELS--

18	Frontal_Sup_L (aal)

13	Left Cerebrum

13	Frontal Lobe

11	brodmann area 6

11	Gray Matter

9	Superior Frontal Gyrus

3	Middle Frontal Gyrus

2	Supp_Motor_Area_L (aal)

>>
