# Supplementary material for: Abnormal coordination of executive control network and default mode network in cognitive impairment after deep nuclei intracerebral hemorrhage
Source: Front Neurol. 2026 Sep 16;17:1915567. doi: 10.3389/fneur.2026.1915567 (PMC13623672; doi:10.3389/fneur.2026.1915567)
Supplement: Supplementary file 6 [file Table_4.docx]

Supplementary Table 4 **Brain detail region of FC with R-DLPFC as ROI positively correlated with cognition**

This report is based on CUI Xu's xjview. (http://www.alivelearn.net/xjview/)

Revised by YAN Chao-Gan and ZHU Wei-Xuan 20091108: suitable for different Cluster Connectivity Criterion: surface connected, edge connected, corner connected.

Number of clusters found: 12

----------------------

Cluster 1

Number of voxels: 56

Peak MNI coordinate: -30 -12 -30

Peak MNI coordinate region: // Left Cerebrum // Limbic Lobe // Parahippocampa Gyrus // White Matter // undefined // Fusiform_L (aal)

Peak intensity: 0.30996

\# voxels	structure

56	--TOTAL # VOXELS--

56	Left Cerebrum

47	White Matter

32	Temporal Lobe

26	Sub-Gyral

22	Fusiform_L (aal)

22	Limbic Lobe

15	Parahippocampa Gyrus

8	ParaHippocampal_L (aal)

8	Temporal_Inf_L (aal)

7	Gray Matter

7	brodmann area 20

7	Uncus

6	Fusiform Gyrus

2	Hippocampus_L (aal)

2	Sub-lobar

2	Lateral Ventricle

2	Cerebro-Spinal Fluid

1	Amygdala_L (aal)

1	Temporal_Mid_L (aal)

----------------------

Cluster 2

Number of voxels: 26

Peak MNI coordinate: -24 -48 -24

Peak MNI coordinate region: // Left Cerebellum // Cerebellum Anterior Lobe // Culmen // undefined // undefined // Cerebelum_4_5_L (aal)

Peak intensity: 0.26957

\# voxels	structure

26	--TOTAL # VOXELS--

22	Left Cerebellum

21	Cerebellum Anterior Lobe

21	Culmen

14	Cerebelum_6_L (aal)

7	Fusiform_L (aal)

5	Cerebelum_4_5_L (aal)

4	Left Cerebrum

4	Temporal Lobe

4	brodmann area 37

4	Gray Matter

2	Fusiform Gyrus

1	Cerebellum Posterior Lobe

1	Declive

----------------------

Cluster 3

Number of voxels: 25

Peak MNI coordinate: -42 -84 -21

Peak MNI coordinate region: // Left Cerebrum // Occipital Lobe // Inferior Occipital Gyrus // Gray Matter // brodmann area 18 // undefined

Peak intensity: 0.29065

\# voxels	structure

25	--TOTAL # VOXELS--

16	Cerebelum_Crus1_L (aal)

13	Occipital Lobe

13	Left Cerebrum

10	Gray Matter

9	Declive

9	Cerebellum Posterior Lobe

9	Left Cerebellum

7	Fusiform Gyrus

6	Inferior Occipital Gyrus

5	brodmann area 19

5	brodmann area 18

3	Fusiform_L (aal)

1	White Matter

----------------------

Cluster 4

Number of voxels: 42

Peak MNI coordinate: -18 -72 -15

Peak MNI coordinate region: // Left Cerebrum // Occipital Lobe // Lingual Gyrus // undefined // undefined // Cerebelum_6_L (aal)

Peak intensity: 0.32345

\# voxels	structure

42	--TOTAL # VOXELS--

34	Left Cerebrum

33	Occipital Lobe

23	Lingual Gyrus

21	Lingual_L (aal)

17	Gray Matter

14	Cerebelum_6_L (aal)

9	brodmann area 19

9	White Matter

7	Cerebellum Posterior Lobe

7	Left Cerebellum

7	Declive

6	brodmann area 18

6	Fusiform_L (aal)

6	Fusiform Gyrus

4	Sub-Gyral

1	Posterior Cingulate

1	Limbic Lobe

1	brodmann area 30

1	Calcarine_L (aal)

----------------------

Cluster 5

Number of voxels: 49

Peak MNI coordinate: 36 42 -9

Peak MNI coordinate region: // Right Cerebrum // Frontal Lobe // Middle Frontal Gyrus // White Matter // undefined // Frontal_Mid_Orb_R (aal)

Peak intensity: 0.2797

\# voxels	structure

49	--TOTAL # VOXELS--

44	Right Cerebrum

43	Frontal Lobe

41	Middle Frontal Gyrus

34	White Matter

25	Frontal_Inf_Orb_R (aal)

24	Frontal_Mid_Orb_R (aal)

6	Gray Matter

5	brodmann area 11

1	brodmann area 47

----------------------

Cluster 6

Number of voxels: 41

Peak MNI coordinate: -63 -18 -9

Peak MNI coordinate region: // Left Cerebrum // Temporal Lobe // Middle Temporal Gyrus // Gray Matter // brodmann area 21 // Temporal_Mid_L (aal)

Peak intensity: 0.3042

\# voxels	structure

41	--TOTAL # VOXELS--

41	Left Cerebrum

41	Temporal Lobe

29	Temporal_Mid_L (aal)

25	Gray Matter

23	Superior Temporal Gyrus

18	Middle Temporal Gyrus

15	brodmann area 21

12	Temporal_Sup_L (aal)

9	White Matter

6	brodmann area 42

3	brodmann area 22

----------------------

Cluster 7

Number of voxels: 55

Peak MNI coordinate: 66 -12 -6

Peak MNI coordinate region: // Right Cerebrum // Temporal Lobe // Middle Temporal Gyrus // Gray Matter // brodmann area 21 // Temporal_Sup_R (aal)

Peak intensity: 0.32273

\# voxels	structure

55	--TOTAL # VOXELS--

39	Temporal Lobe

39	Temporal_Sup_R (aal)

39	Right Cerebrum

25	Middle Temporal Gyrus

21	Gray Matter

15	brodmann area 21

14	Superior Temporal Gyrus

10	Temporal_Mid_R (aal)

8	White Matter

6	Temporal_Pole_Sup_R (aal)

4	brodmann area 22

2	brodmann area 38

----------------------

Cluster 8

Number of voxels: 99

Peak MNI coordinate: -36 60 -6

Peak MNI coordinate region: // Left Cerebrum // Frontal Lobe // Middle Frontal Gyrus // Gray Matter // brodmann area 10 // Frontal_Mid_Orb_L (aal)

Peak intensity: 0.34302

\# voxels	structure

99	--TOTAL # VOXELS--

91	Left Cerebrum

89	Frontal Lobe

64	Middle Frontal Gyrus

42	White Matter

37	Frontal_Mid_Orb_L (aal)

34	Frontal_Inf_Orb_L (aal)

34	Gray Matter

16	brodmann area 10

13	Inferior Frontal Gyrus

12	brodmann area 11

10	Superior Frontal Gyrus

7	Frontal_Inf_Tri_L (aal)

6	brodmann area 47

4	Frontal_Mid_L (aal)

1	Frontal_Sup_Orb_L (aal)

----------------------

Cluster 9

Number of voxels: 19

Peak MNI coordinate: 60 -33	3

Peak MNI coordinate region: // Right Cerebrum // Temporal Lobe // Middle Temporal Gyrus // Gray Matter // brodmann area 22 // Temporal_Mid_R (aal)

Peak intensity: 0.26664

\# voxels	structure

19	--TOTAL # VOXELS--

19	Right Cerebrum

19	Temporal Lobe

15	Middle Temporal Gyrus

15	Temporal_Mid_R (aal)

11	White Matter

8	Gray Matter

5	brodmann area 22

4	Temporal_Sup_R (aal)

4	Superior Temporal Gyrus

2	brodmann area 21

----------------------

Cluster 10

Number of voxels: 83

Peak MNI coordinate: -9 -87	6

Peak MNI coordinate region: // Left Cerebrum // Occipital Lobe // Cuneus // White Matter // undefined // Calcarine_L (aal)

Peak intensity: 0.3029

\# voxels	structure

83	--TOTAL # VOXELS--

77	Occipital Lobe

77	Left Cerebrum

38	Lingual Gyrus

37	Cuneus

32	White Matter

31	Lingual_L (aal)

30	Calcarine_L (aal)

28	Gray Matter

22	brodmann area 18

6	Occipital_Sup_L (aal)

5	Cerebelum_Crus1_L (aal)

4	Cerebelum_6_L (aal)

4	brodmann area 17

3	Cuneus_L (aal)

2	Middle Occipital Gyrus

1	Inter-Hemispheric

----------------------

Cluster 11

Number of voxels: 34

Peak MNI coordinate: -51 18 30

Peak MNI coordinate region: // Left Cerebrum // Frontal Lobe // Middle Frontal Gyrus // White Matter // undefined // Frontal_Inf_Tri_L (aal)

Peak intensity: 0.32976

\# voxels	structure

34	--TOTAL # VOXELS--

34	Frontal Lobe

34	Left Cerebrum

24	Middle Frontal Gyrus

17	Gray Matter

17	White Matter

15	brodmann area 9

14	Precentral_L (aal)

11	Frontal_Inf_Tri_L (aal)

10	Inferior Frontal Gyrus

5	Frontal_Inf_Oper_L (aal)

4	Frontal_Mid_L (aal)

2	brodmann area 46

----------------------

Cluster 12

Number of voxels: 39

Peak MNI coordinate: -33 -48 57

Peak MNI coordinate region: // Left Cerebrum // Parietal Lobe // Inferior Parietal Lobule // White Matter // undefined // Parietal_Sup_L (aal)

Peak intensity: 0.29822

\# voxels	structure

39	--TOTAL # VOXELS--

34	Parietal Lobe

34	Left Cerebrum

30	Inferior Parietal Lobule

21	Parietal_Inf_L (aal)

18	White Matter

16	Gray Matter

15	brodmann area 40

12	Parietal_Sup_L (aal)

3	Postcentral_L (aal)

3	Postcentral Gyrus

1	brodmann area 7

1	Superior Parietal Lobule

>>
