# Supplementary material for: Abnormal coordination of executive control network and default mode network in cognitive impairment after deep nuclei intracerebral hemorrhage
Source: Front Neurol. 2026 Sep 16;17:1915567. doi: 10.3389/fneur.2026.1915567 (PMC13623672; doi:10.3389/fneur.2026.1915567)
Supplement: Supplementary file 7 [file Table_5.docx]

Supplementary Table 5 **Brain detail regions of FC with PCC as ROI positively and negatively correlated with cognition**

This report is based on CUI Xu's xjview. (http://www.alivelearn.net/xjview/)

Revised by YAN Chao-Gan and ZHU Wei-Xuan 20091108: suitable for different Cluster Connectivity Criterion: surface connected, edge connected, corner connected.

Number of clusters found: 18

----------------------

Cluster 1

Number of voxels: 39

Peak MNI coordinate: 51 -66 -45

Peak MNI coordinate region: // Right Cerebellum // Cerebellum Posterior Lobe // Inferior Semi-Lunar Lobule // undefined // undefined // Cerebelum_Crus2_R (aal)

Peak intensity: 0.31695

\# voxels	structure

39	--TOTAL # VOXELS--

36	Right Cerebellum

36	Cerebellum Posterior Lobe

32	Cerebelum_Crus2_R (aal)

21	Inferior Semi-Lunar Lobule

13	Pyramis

3	Cerebelum_7b_R (aal)

2	Tuber

1	Cerebelum_8_R (aal)

----------------------

Cluster 2

Number of voxels: 36

Peak MNI coordinate: -15 -33 -51

Peak MNI coordinate region: // undefined // undefined // undefined // undefined // undefined // undefined

Peak intensity: 0.30914

\# voxels	structure

36	--TOTAL # VOXELS--

19	Cerebelum_9_L (aal)

11	Cerebellar Tonsil

11	Left Cerebellum

11	Cerebellum Posterior Lobe

----------------------

Cluster 3

Number of voxels: 35

Peak MNI coordinate: 15 -45 -51

Peak MNI coordinate region: // Right Cerebellum // Cerebellum Posterior Lobe // Cerebellar Tonsil // undefined // undefined // Cerebelum_9_R (aal)

Peak intensity: 0.27685

\# voxels	structure

35	--TOTAL # VOXELS--

29	Cerebelum_9_R (aal)

24	Cerebellar Tonsil

24	Right Cerebellum

24	Cerebellum Posterior Lobe

1	Cerebelum_8_R (aal)

----------------------

Cluster 4

Number of voxels: 223

Peak MNI coordinate: 57	3 -24

Peak MNI coordinate region: // Right Cerebrum // Temporal Lobe // Middle Temporal Gyrus // Gray Matter // brodmann area 21 // Temporal_Mid_R (aal)

Peak intensity: 0.30834

\# voxels	structure

223	--TOTAL # VOXELS--

189	Right Cerebrum

164	Temporal Lobe

91	White Matter

73	Fusiform_R (aal)

72	Gray Matter

60	Middle Temporal Gyrus

54	Temporal_Mid_R (aal)

50	Fusiform Gyrus

35	Temporal_Pole_Mid_R (aal)

33	Temporal_Inf_R (aal)

30	Superior Temporal Gyrus

26	brodmann area 21

26	Limbic Lobe

23	brodmann area 20

22	Parahippocampa Gyrus

16	Temporal_Pole_Sup_R (aal)

14	Sub-Gyral

10	brodmann area 38

10	brodmann area 36

9	Inferior Temporal Gyrus

4	Cerebellum Anterior Lobe

4	Right Cerebellum

4	Cerebelum_6_R (aal)

4	Culmen

4	ParaHippocampal_R (aal)

4	Uncus

3	brodmann area 37

1	Cerebelum_4_5_R (aal)

----------------------

Cluster 5

Number of voxels: 34

Peak MNI coordinate: -39 12 -42

Peak MNI coordinate region: // undefined // undefined // undefined // undefined // undefined // Temporal_Inf_L (aal)

Peak intensity: 0.27411

\# voxels	structure

34	--TOTAL # VOXELS--

21	Temporal Lobe

21	Left Cerebrum

14	Superior Temporal Gyrus

10	Temporal_Pole_Mid_L (aal)

10	Temporal_Inf_L (aal)

9	Gray Matter

8	brodmann area 38

7	Middle Temporal Gyrus

6	White Matter

1	brodmann area 21

----------------------

Cluster 6

Number of voxels: 70

Peak MNI coordinate: -21 -27 -27

Peak MNI coordinate region: // Left Cerebellum // Cerebellum Anterior Lobe // Culmen // undefined // undefined // Cerebelum_4_5_L (aal)

Peak intensity: 0.40115

\# voxels	structure

70	--TOTAL # VOXELS--

36	Left Cerebellum

36	Cerebellum Anterior Lobe

35	Culmen

32	Pons

32	Left Brainstem

31	Cerebelum_4_5_L (aal)

7	Cerebelum_6_L (aal)

----------------------

Cluster 7

Number of voxels: 82

Peak MNI coordinate: -54 -3 -6

Peak MNI coordinate region: // Left Cerebrum // Temporal Lobe // Superior Temporal Gyrus // Gray Matter // brodmann area 22 // Temporal_Sup_L (aal)

Peak intensity: 0.31674

\# voxels	structure

82	--TOTAL # VOXELS--

82	Left Cerebrum

82	Temporal Lobe

51	White Matter

39	Temporal_Mid_L (aal)

37	Superior Temporal Gyrus

36	Temporal_Sup_L (aal)

32	Middle Temporal Gyrus

29	Gray Matter

14	brodmann area 21

9	brodmann area 22

7	Inferior Temporal Gyrus

7	Temporal_Inf_L (aal)

6	Fusiform Gyrus

5	brodmann area 20

----------------------

Cluster 8

Number of voxels: 26

Peak MNI coordinate: -30	6 -27

Peak MNI coordinate region: // Left Cerebrum // Temporal Lobe // Superior Temporal Gyrus // White Matter // undefined // Temporal_Pole_Sup_L (aal)

Peak intensity: 0.26263

\# voxels	structure

26	--TOTAL # VOXELS--

26	Left Cerebrum

23	White Matter

19	Temporal Lobe

10	Superior Temporal Gyrus

9	Sub-Gyral

7	Limbic Lobe

7	Uncus

5	Temporal_Pole_Sup_L (aal)

4	Amygdala_L (aal)

3	Gray Matter

2	brodmann area 28

1	Temporal_Pole_Mid_L (aal)

1	ParaHippocampal_L (aal)

1	Hippocampus_L (aal)

1	Fusiform_L (aal)

1	Amygdala

1	Temporal_Mid_L (aal)

----------------------

Cluster 9

Number of voxels: 28

Peak MNI coordinate: 39 30 -15

Peak MNI coordinate region: // Right Cerebrum // Frontal Lobe // Inferior Frontal Gyrus // undefined // undefined // Frontal_Inf_Orb_R (aal)

Peak intensity: 0.27165

\# voxels	structure

28	--TOTAL # VOXELS--

27	Right Cerebrum

24	Frontal Lobe

24	Frontal_Inf_Orb_R (aal)

15	Inferior Frontal Gyrus

7	Gray Matter

7	Middle Frontal Gyrus

6	White Matter

4	brodmann area 11

3	brodmann area 47

3	Frontal_Mid_Orb_R (aal)

----------------------

Cluster 10

Number of voxels: 654

Peak MNI coordinate: 3 54 -3

Peak MNI coordinate region: // Right Cerebrum // Frontal Lobe // Medial Frontal Gyrus // Gray Matter // brodmann area 10 // Frontal_Med_Orb_R (aal)

Peak intensity: 0.33514

\# voxels	structure

654	--TOTAL # VOXELS--

393	Frontal Lobe

314	Left Cerebrum

310	Medial Frontal Gyrus

294	White Matter

284	Right Cerebrum

267	Gray Matter

186	Limbic Lobe

172	Anterior Cingulate

157	Frontal_Sup_Medial_R (aal)

139	Frontal_Sup_Medial_L (aal)

128	Cingulum_Ant_L (aal)

89	brodmann area 9

82	brodmann area 10

77	Cingulum_Ant_R (aal)

72	Superior Frontal Gyrus

65	brodmann area 32

29	Inter-Hemispheric

25	Frontal_Sup_R (aal)

22	Frontal_Sup_L (aal)

20	Corpus Callosum

20	Frontal_Med_Orb_R (aal)

19	Sub-lobar

18	Extra-Nuclear

16	brodmann area 24

14	Frontal_Med_Orb_L (aal)

11	Cingulate Gyrus

7	Middle Frontal Gyrus

6	brodmann area 8

6	Frontal_Mid_R (aal)

6	brodmann area 6

5	Cingulum_Mid_R (aal)

4	Sub-Gyral

2	brodmann area 33

1	Lateral Ventricle

1	Cerebro-Spinal Fluid

----------------------

Cluster 11

Number of voxels: 30

Peak MNI coordinate: -27 -57 21

Peak MNI coordinate region: // Left Cerebrum // Temporal Lobe // Sub-Gyral // White Matter // undefined // undefined

Peak intensity: -0.34902

\# voxels	structure

30	--TOTAL # VOXELS--

30	Left Cerebrum

29	White Matter

26	Sub-Gyral

14	Temporal Lobe

11	Parietal Lobe

2	Limbic Lobe

2	Frontal Lobe

2	Cingulate Gyrus

1	Sub-lobar

1	Middle Temporal Gyrus

1	Extra-Nuclear

1	brodmann area 31

1	Gray Matter

----------------------

Cluster 12

Number of voxels: 167

Peak MNI coordinate: 42 33 30

Peak MNI coordinate region: // Right Cerebrum // Frontal Lobe // Middle Frontal Gyrus // White Matter // undefined // Frontal_Mid_R (aal)

Peak intensity: 0.31105

\# voxels	structure

167	--TOTAL # VOXELS--

167	Frontal Lobe

167	Right Cerebrum

133	Middle Frontal Gyrus

89	White Matter

86	Frontal_Mid_R (aal)

77	Frontal_Inf_Tri_R (aal)

67	Gray Matter

30	brodmann area 9

29	Inferior Frontal Gyrus

26	brodmann area 46

7	brodmann area 45

5	Superior Frontal Gyrus

2	brodmann area 10

1	Frontal_Inf_Oper_R (aal)

----------------------

Cluster 13

Number of voxels: 28

Peak MNI coordinate: 60	6 27

Peak MNI coordinate region: // Right Cerebrum // Frontal Lobe // Inferior Frontal Gyrus // White Matter // undefined // Precentral_R (aal)

Peak intensity: 0.29141

\# voxels	structure

28	--TOTAL # VOXELS--

28	Frontal Lobe

28	Right Cerebrum

22	Inferior Frontal Gyrus

18	Precentral_R (aal)

13	White Matter

10	Gray Matter

9	Frontal_Inf_Oper_R (aal)

7	brodmann area 9

6	Precentral Gyrus

2	brodmann area 6

1	brodmann area 45

1	Postcentral_R (aal)

----------------------

Cluster 14

Number of voxels: 34

Peak MNI coordinate: -51 21 33

Peak MNI coordinate region: // Left Cerebrum // Frontal Lobe // Middle Frontal Gyrus // White Matter // undefined // Frontal_Inf_Oper_L (aal)

Peak intensity: 0.26039

\# voxels	structure

34	--TOTAL # VOXELS--

34	Frontal Lobe

34	Left Cerebrum

34	Middle Frontal Gyrus

25	Frontal_Mid_L (aal)

21	White Matter

12	Gray Matter

9	brodmann area 9

5	Frontal_Inf_Oper_L (aal)

4	Frontal_Inf_Tri_L (aal)

3	brodmann area 8

----------------------

Cluster 15

Number of voxels: 344

Peak MNI coordinate: -36 -78 45

Peak MNI coordinate region: // undefined // undefined // undefined // undefined // undefined // undefined

Peak intensity: 0.31892

\# voxels	structure

344	--TOTAL # VOXELS--

271	Left Cerebrum

249	Parietal Lobe

137	Gray Matter

130	Parietal_Inf_L (aal)

113	Postcentral_L (aal)

109	Postcentral Gyrus

106	White Matter

102	Inferior Parietal Lobule

65	brodmann area 40

24	brodmann area 2

22	Frontal Lobe

22	Sub-Gyral

18	Precentral Gyrus

17	Parietal_Sup_L (aal)

15	Angular_L (aal)

13	brodmann area 7

13	brodmann area 3

11	Precuneus

9	brodmann area 4

9	SupraMarginal_L (aal)

8	Superior Parietal Lobule

5	brodmann area 19

4	brodmann area 6

4	brodmann area 1

4	Precentral_L (aal)

1	Occipital_Mid_L (aal)

1	Middle Frontal Gyrus

----------------------

Cluster 16

Number of voxels: 22

Peak MNI coordinate: -3 -72 48

Peak MNI coordinate region: // Left Cerebrum // Parietal Lobe // Precuneus // Gray Matter // brodmann area 7 // Precuneus_L (aal)

Peak intensity: 0.25147

\# voxels	structure

22	--TOTAL # VOXELS--

16	Precuneus_L (aal)

16	Precuneus

14	Parietal Lobe

13	Left Cerebrum

9	brodmann area 7

9	Gray Matter

5	White Matter

4	Inter-Hemispheric

2	Occipital_Sup_L (aal)

1	Right Cerebrum

1	Precuneus_R (aal)

----------------------

Cluster 17

Number of voxels: 37

Peak MNI coordinate: 30 -69 60

Peak MNI coordinate region: // undefined // undefined // undefined // undefined // undefined // Parietal_Sup_R (aal)

Peak intensity: 0.28288

\# voxels	structure

37	--TOTAL # VOXELS--

25	Angular_R (aal)

13	Right Cerebrum

13	Parietal Lobe

12	Inferior Parietal Lobule

9	Gray Matter

8	brodmann area 40

7	Parietal_Sup_R (aal)

4	Parietal_Inf_R (aal)

2	White Matter

1	brodmann area 7

1	Superior Parietal Lobule

----------------------

Cluster 18

Number of voxels: 125

Peak MNI coordinate: 36 24 54

Peak MNI coordinate region: // Right Cerebrum // Frontal Lobe // Superior Frontal Gyrus // Gray Matter // brodmann area 8 // Frontal_Mid_R (aal)

Peak intensity: 0.28151

\# voxels	structure

125	--TOTAL # VOXELS--

111	Right Cerebrum

111	Frontal Lobe

69	Frontal_Mid_R (aal)

68	Middle Frontal Gyrus

47	Gray Matter

44	White Matter

38	brodmann area 8

36	Superior Frontal Gyrus

27	Frontal_Sup_R (aal)

7	brodmann area 6

2	Sub-Gyral

2	brodmann area 9

2	Precentral_R (aal)

1	Medial Frontal Gyrus

>>
